# A metabolomics cell-based approach for anticipating and investigating drug-induced liver injury

**DOI:** 10.1038/srep27239

**Published:** 2016-06-06

**Authors:** Juan Carlos García- Cañaveras, José V. Castell, M. Teresa Donato, Agustín Lahoz

**Affiliations:** 1 Unidad de Hepatología Experimental, Instituto de Investigación Sanitaria - Fundación Hospital La Fe, Valencia, Spain. CIBERehd, Centro de Investigaciones Biomédicas en Red de Enfermedades Hepáticas y Digestivas, FIS, Spain; 2 Departamento de Bioquímica y Biología Molecular, Facultad de Medicina, Universidad de Valencia, Spain

## Abstract

In preclinical stages of drug development, anticipating potential adverse drug effects such as toxicity is an important issue for both saving resources and preventing public health risks. Current *in vitro* cytotoxicity tests are restricted by their predictive potential and their ability to provide mechanistic information. This study aimed to develop a metabolomic mass spectrometry-based approach for the detection and classification of drug-induced hepatotoxicity. To this end, the metabolite profiles of human derived hepatic cells (i.e., HepG2) exposed to different well-known hepatotoxic compounds acting through different mechanisms (i.e., oxidative stress, steatosis, phospholipidosis, and controls) were compared by multivariate data analysis, thus allowing us to decipher both common and mechanism-specific altered biochemical pathways. Briefly, oxidative stress damage markers were found in the three mechanisms, mainly showing altered levels of metabolites associated with glutathione and γ-glutamyl cycle. Phospholipidosis was characterized by a decreased lysophospholipids to phospholipids ratio, suggestive of phospholipid degradation inhibition. Whereas, steatosis led to impaired fatty acids β-oxidation and a subsequent increase in triacylglycerides synthesis. The characteristic metabolomic profiles were used to develop a predictive model aimed not only to discriminate between non-toxic and hepatotoxic drugs, but also to propose potential drug toxicity mechanism(s).

Drug-induced liver injury (DILI) is a health problem that poses an important challenge for clinicians, the pharmaceutical industry and regulatory agencies^1^. DILI is a complex phenomenon which encompasses a wide spectrum of clinical manifestations (from mild biochemical alterations to acute liver failure) and represents the most frequent cause of acute liver failure^2^,^3^. Hepatotoxicity is also a major safety issue in drug development and is a leading cause of attrition of drug candidates, restriction of use and post-market withdrawal of approved drugs^1^,^4^. Safety assays during drug development are performed to minimize potential risks to humans and reduce financial costs. Preclinical testing in laboratory animals often fails to predict human DILI because of the major interspecies differences in drug metabolism and toxicity targets^5^. In this scenario, human liver-derived cells constitute valuable models for *in vitro* hepatotoxicity screenings^6^. Their suitability for investigating the molecular and cellular processes involved in hepatotoxicity and their abilities to detect potential toxic effects before drug candidates are tested in animals and enter in clinical trials have been amply demonstrated^6^.

Traditionally, *in vitro* toxicity screenings have relied on the use of single-endpoint measurements aimed to estimate cell viability and/or the functional metabolic state of cells previously exposed to test compounds. These assays usually monitor events that occur late in the cell injury process^6^,^7^, and have unfortunately shown poor prediction of human hepatotoxicity^6^,^8^,^9^. Therefore, the development of reliable screening approaches able to detect hepatotoxicity early in the drug development remains a challenge. With the advent of the *“omics”* technologies, new approaches have been developed to propose predictive signatures and to study drug toxicity mechanisms^10^,^11^,^12^. The simultaneous measurement of multiple parameters may contribute to the development of more accurate and predictive strategies^13^. Multiparametric approaches integrate data obtained simultaneously from different cell function indicators, which may suggest the mechanism of toxic action of a given compound and help in prioritizing compounds in drug discovery based on their potential hepatotoxicity to humans. In this sense, transcriptomic-based analyses or cell imaging technology have been proposed for hepatotoxicity screenings in cultured cells^11^,^14^,^15^. Although these assays offer the possibility of detecting subtle toxicity-related changes that may go unnoticed with mono-parametric assays^8^, they fail to provide translational biomarkers and report limited mechanistic information from a functional point of view. Metabolomics, which is aimed to the unbiased measurement of all the “downstream” products of genes and proteins (i.e., metabolites), could complete the mechanistic information provided by other *“omics”* and imaging approaches^16^. The capabilities of metabolomics to assess the cell response to external stimuli have been widely demonstrated, several studies reported their use in biomarkers discovery and providing new insights into drug modes of action^12^,^17^,^18^. Metabolomics provides the closest information to the phenotype of the system under study (cell, tissue, and organism), which, in the case of patients, could be used to obtain new toxicity-related biomarkers easily amenable to the clinic^19^.

In the present study, we assessed the capabilities of liquid chromatography (LC) coupled to mass spectrometry (MS) based untargeted metabolite profiling as tool to detect and classify the potential hepatotoxicity of new drugs. To this end, the metabolite profiles of HepG2 cells, which were previously exposed to different well-known model compounds that induce hepatotoxicity through different mechanisms (i.e., oxidative stress, steatosis, phospholipidosis, and controls), were obtained. Then, multivariate data analysis was used for the differential comparison of these metabolite profiles and to decipher discriminant mechanism-specific metabolic signatures. Such specific metabolomic fingerprints were used to develop a predictive model aimed not only to discriminate between non-toxic and hepatotoxic drugs but also to propose their main toxicity mechanism(s). In addition, the rich metabolome data was submitted to functional enrichment analysis that allowed us to unravel those cellular pathways most significantly altered in each toxic mechanism.

## Results

### Metabolite profiling of drug-induced hepatotoxicity

**Figure 1:**
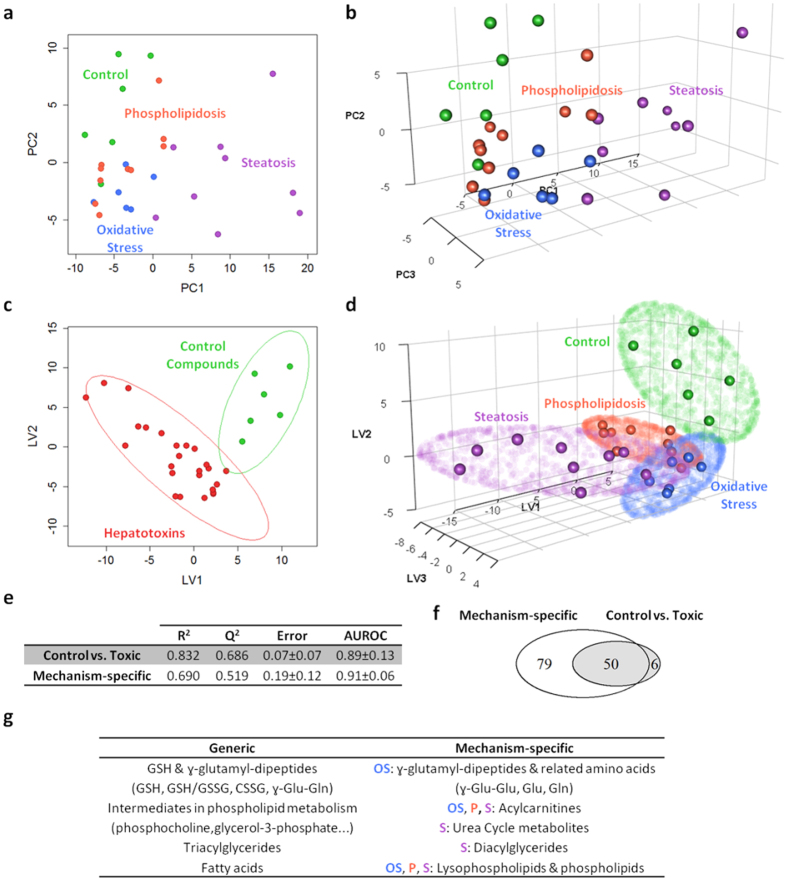
Multivariate data analysis overview of the metabolomic changes induced by the model toxic compounds. PCA scores plots performed using two (**a**) or three (**b**) principal components corresponding to data obtained from HepG2 cells treated with hepatotoxins acting through different mechanisms. Each point summarizes all the information provided by the four different analytical conditions (272 identified metabolites). (**c**) PLS-DA scores plot corresponding to a model built using two latent variables and aimed at the discrimination between control compounds and hepatotoxicants. The lines denote 95% confidence interval Hotelling’s ellipses. (**d**) PLS-DA scores plot corresponding to a model built using three latent variables and aimed at the discrimination among the different mechanisms of hepatotoxicity. The lines denote 95% confidence interval Hotelling’s ellipses. (**e**) Figures of merit of the PLS-DA models. Misclassification error and AUROC are expressed as mean ± standard deviation. (**f**) Venn diagram showing the overlap among the metabolites altered by the control vs. toxic analysis with respect to the analysis focused on a mechanism-specific discrimination. (**g**) Differential metabolites/metabolic pathways/classes of metabolites alterations detected using either the control vs. toxic or the mechanism-specific-based discrimination. OS: oxidative stress; P: phospholipidosis; S: steatosis.

HepG2 cells were exposed for 24 h to non-lethal concentrations of model hepatotoxins and non-toxic drugs (Table 1). Cell monolayers were then processed and subsequently analyzed following a previously described LC-MS-based untargeted metabolic profiling strategy, which allowed for the determination of more than 300 metabolites comprising both polar and non-polar compounds^20^,^21^. First, LC-MS data quality was assessed by using several internal standards (IS) and quality control (QC) samples (Supplementary Table S1). Then, data sets were subjected to non-supervised multivariate data analysis techniques to evaluate the presence of any metabolomic pattern that could discriminate between toxic and non-toxic compounds. The PCA (principal component analysis) scores plots showed an almost complete separation between cells treated with control compounds and those treated with hepatotoxic compounds, while the differences among the three toxicity mechanisms were not so clear (Fig. 1a,b). To evaluate whether the analytical strategy was able to distinguish between those alterations that may be considered as common to all the toxic events from those more mechanism-specific, a supervised analysis of the MS-data was performed considering groups on the following bases; toxic vs non-toxic (Fig. 1c) and mechanism-based groups (Fig. 1d). Both approaches led to a clear separation of the classes as shown in the PLS-DA (projection to latent structures-discriminant analysis) scores plots, and all the models showed good figures of merit for the PLS-DA, based on cross-validation (Fig. 1e). A detailed inspection of the discriminant metabolites revealed that although some of them were present in both analyses, a set of metabolites remained mechanism-specific (Fig. 1f). Information of the common and mechanism-specific altered metabolites and metabolites classes is provided in Fig. 1g.

### Unraveling toxicity-related pathways

**Figure 2:**
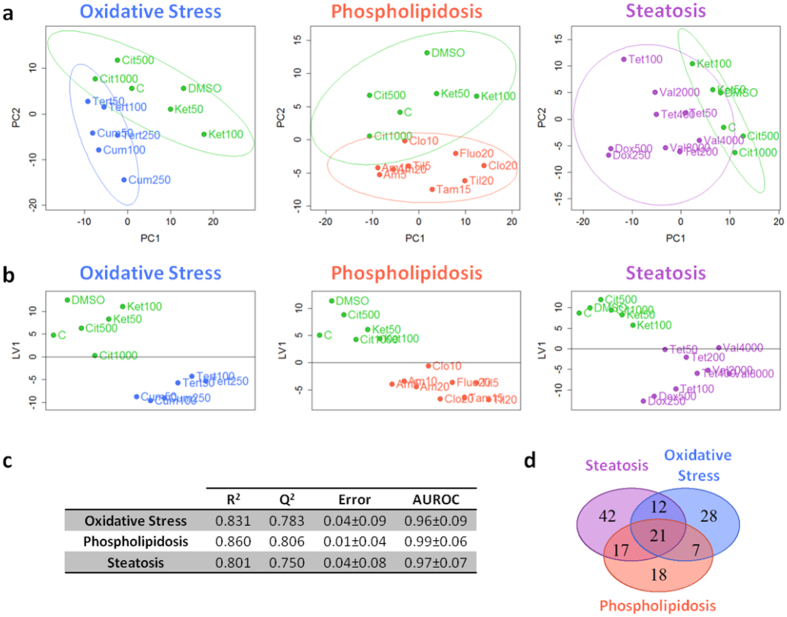
Multivariate data analysis of the metabolomic changes induced by each mechanism of hepatotoxicity. PCA (**a**) and PLS-DA (**b**) scores plots corresponding to pairwise comparisons (i.e. control vs. each mechanism of hepatotoxicity) of the data obtained from HepG2 cells treated with hepatotoxins acting through different mechanisms. Each point summarizes all the information provided by the four different analytical conditions (272 identified metabolites). The lines denote 95% confidence interval Hotelling’s ellipses. PCA models were developed using two principal components. PLS-DA models were built using one latent variable. (**c**) Figures of merit of the PLS-DA models. Misclassification error and AUROC are expressed as mean ± standard deviation. (**d**) Venn diagram showing the overlap among the metabolites altered by the model compounds representative of different toxicity mechanisms. Green: control; blue: oxidative stress; red: phospholipidosis; purple: steatosis. Abbreviations corresponding to drug names and concentrations are depicted in Table 1, C corresponds to control culture and DMSO to control culture with DMSO at 0.5% (v/v).

To highlight the differences induced by each hepatotoxicity mechanism, pairwise comparisons (with respect to non-toxic compounds) were performed. A clear separation between the cells treated with non-hepatotoxic compounds (controls) and those treated with the different hepatotoxic model compounds (i.e., oxidative stress, phospholipidosis and steatosis) was observed by the use of non-supervised data analys (i.e. PCA) (Fig. 2a). Noticeable and specific metabolomic changes for each group of compounds was observed, so uni- and multivariate supervised data analysis techniques were employed to identify those metabolites that were significantly altered because of each specific toxicity mechanism (Fig. 2b,c). The analysis revealed the presence of both common and mechanism-specific altered metabolites (Fig. 2d). However, the magnitude or even the direction of the change was significantly different for many of them (Supplementary Tables S2 and S3). The specific set of discriminant metabolites found in each pairwise comparison were submitted to metabolic pathway enrichment analysis to map the pathways and cell functions most significantly altered. Major mechanism-specific results are summarized below.

### Oxidative stress inducers

**Figure 3:**
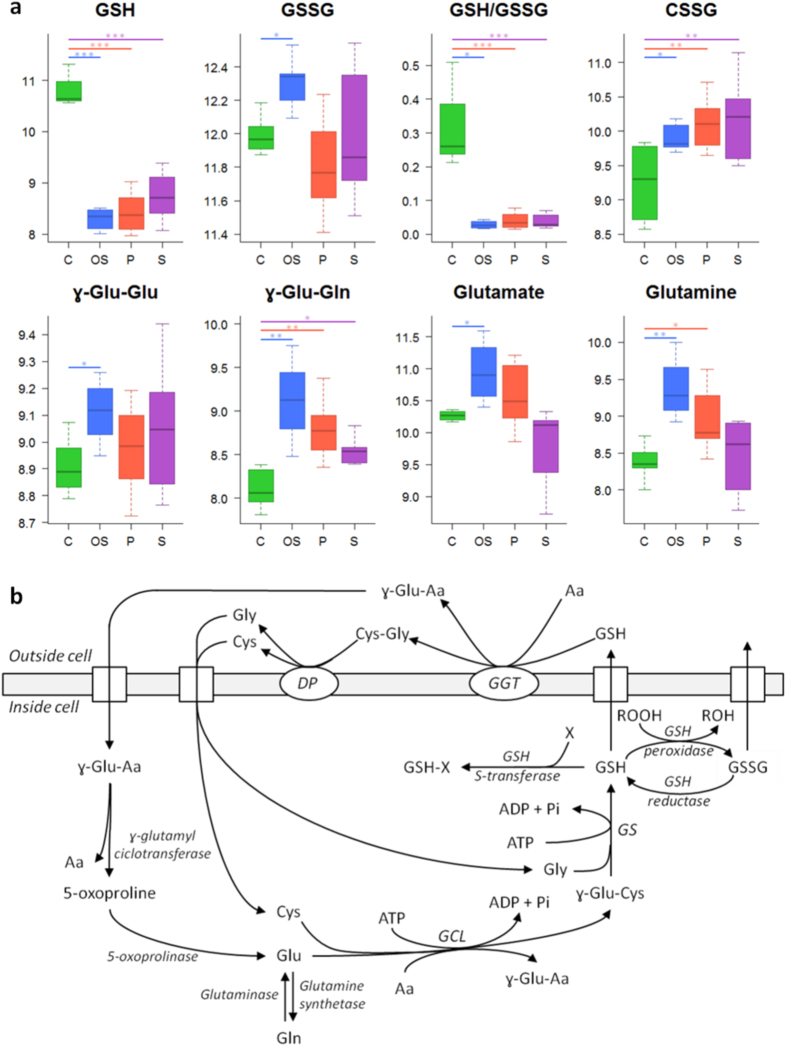
Metabolic alterations related to GSH metabolism and the γ-glutamyl cycle. (**a**) Boxplots showing the altered metabolites. Boxes denote interquartile ranges, lines denote medians and whiskers denote the 10^th^ and 90^th^ percentiles. Metabolite relative abundance, expressed as ratio or log-transformed, is calculated by referring metabolite peak intensity to a constant concentration of internal standard per mg of tissue. Green: control (C); blue: oxidative stress (OS); red: phospholipidosis (P); purple: steatosis (S). *, *q* value < 0.05; **, *q* value < 0.01; ***, *q* value < 0.001 calculated using the Mann Whitney test corrected for multiple testing by using FDR. (**b)** The γ-glutamyl cycle, which accounts for GSH synthesis and recycling. Enzymes are denoted in italics. Square boxes denote transmembrane transporters. Aa: amino acid; Cys: cysteine; Cys-Gly: cysteinyl-glycine; DP: dipeptidase; GCL: glutamate cysteine ligase; γ-Glu-Aa: γ-glutamyl amino acid; GGT: γ-glutamyl transpeptidase; Glu: glutamate; Gly: glycine; GS: glutathione synthetase.

**Figure 4:**
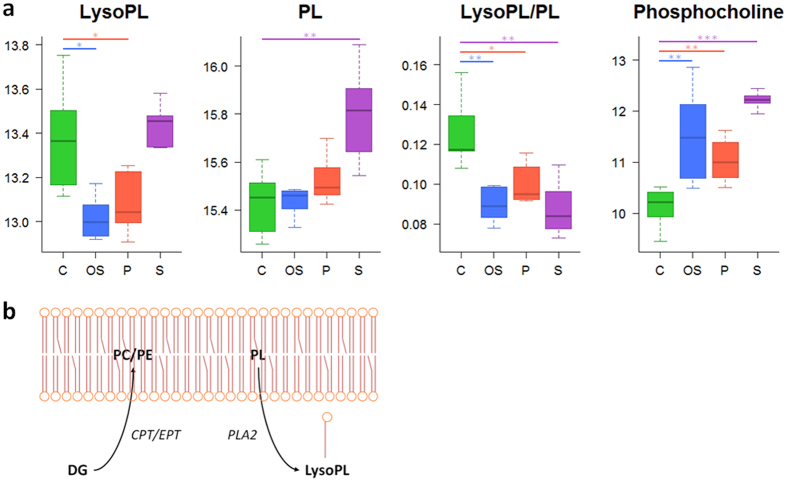
Metabolic alterations related to phospholipid metabolism. (**a)** Boxplots showing the altered metabolites. Boxes denote interquartile ranges, lines denote medians and whiskers denote the 10^th^ and 90^th^ percentiles. Metabolite relative abundance, expressed as ratio or log-transformed, is calculated by referring metabolite peak intensity to a constant concentration of internal standard per mg of tissue. Green: control (C); blue: oxidative stress (OS); red: phospholipidosis (P); purple: steatosis (S). *, *q* value < 0.05; **, *q* value < 0.01; ***, *q* value < 0.001 calculated using the Mann Whitney test corrected for multiple testing by using FDR. (**b**) Phospholipid synthesis, via the Kennedy pathway, and degradation, through the action of phospholipase A2 (PLA2). PC: phosphatidylcholine; PE: phospohatidylethanolamine; phospholipid, DG: diacylglyceride; CPT: 1,2-diacylglycerol cholinephosphotransferase; EPT: 1,2-diacylglycerol ethanolaminephosphotransferase.

**Figure 5:**
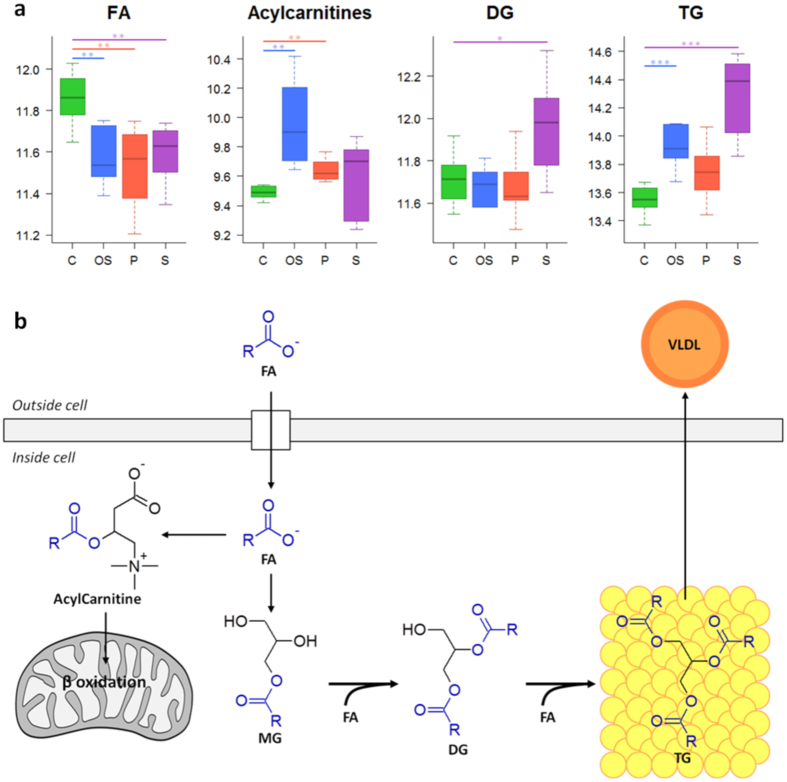
Metabolic alterations related to fatty acids and triacylglycerides metabolism. (**a)** Boxplots showing the altered metabolites. Boxes denote interquartile ranges, lines denote medians and whiskers denote 10th and 90th percentiles. Metabolite relative abundance, expressed as ratio or log-transformed, is calculated by referring metabolite peak intensity to a constant concentration of internal standard per mg of tissue. Green: control (C); blue: oxidative stress (OS); red: phospholipidosis (P); purple: steatosis (S). *, *q* value < 0.05; **, *q* value < 0.01; ***, *q* value < 0.001 calculated using the Mann Whitney test corrected for multiple testing by using FDR. (**b)** Fatty acids and triacylglycerides metabolism in the liver. DG: diacylglyceride; FA: fatty acid; MG: monoacylglyceride; TG: triacylglyceride; VLDL: very low-density lioprotein.

A total of 68 metabolites were significantly altered after exposure of HepG2 cells to oxidative stress inducers (Supplementary Table S2). As expected, alterations in well-known low-molecular-weight oxidative stress markers (i.e., reduced glutathione (GSH), oxidized glutathione (GSSG), cysteine glutathione disulfide (CSSG), γ-glutamyl dipeptides, glutamate and glutamine) were found (Fig. 3). Pathway enrichment analysis showed that glutamate, GSH, nitrogen, amino acid and nucleobases metabolism pathways were altered after the toxic insult. Additionally, alterations in lipid homeostasis covering fatty acid (FA), triacylglyceride (TG) and phospholipid (PL) metabolism were also detected (Figs 4 and 5).

### Phospholipidogenic drugs

Phospholipidogenic drugs induced changes in 63 identified metabolites (Supplementary Table S2). The pathway analysis showed PL, unsaturated FA metabolism, urea cycle, glutamate, and GSH metabolism as the most significantly altered biochemical pathways. Although, no notable changes were observed for both PL and LysoPL levels, a significant decrease in the LysoPL/PL ratio was detected (Fig. 4, Supplementary Table S3). With respect to polar metabolites, the most remarkable changes were associated with the appearance of oxidative stress markers (i.e. increased levels of CSSG and decreased levels of GSH and the GSH/GSSG ratio) (Fig. 3).

### Steatogenic drugs

Drug-induced steatosis in HepG2 cells resulted in the significant alteration of 92 metabolites (Supplementary Table S2). A functional enrichment analysis pointed FA, TG, amino acid, urea cycle, nitrogen, PL, glutamate, cysteine and GSH metabolism as the most affected pathways. The main lipidome alterations were the increase in the levels of TG, diacylglycerides (DG), PL and LysoPL and a decrease in FA levels (Figs 4 and 5). With respect to polar metabolites, although several compounds were altered as a result of drug-induced steatosis, it is worth noting the significant changes observed in the oxidative stress markers (Fig. 3).

### Development and validation of a hepatotoxicity predictive model

**Figure 6:**
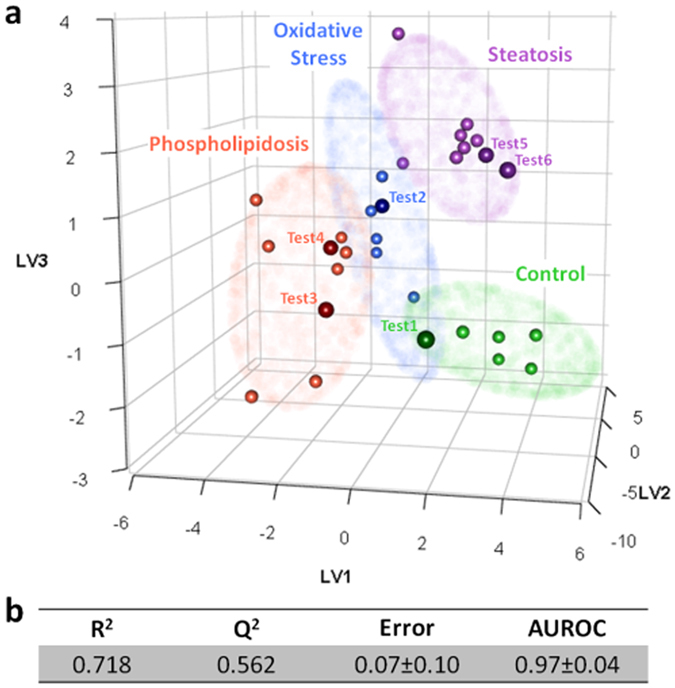
Overview of the PLS-DA model aimed at the discrimination among the different mechanisms of hepatotoxicity. The PLS-DA model was built using three latent variables and the top- 26 ranked variables based on the model development subset with data obtained from the metabolomic analysis of HepG2 treated with either non-toxic or hepatotoxic compounds acting through different mechanism of hepatotoxicity (i.e. oxidative stress, phospholipidosis and steatosis). (**a)** Scores plot. The lines denote 95% confidence interval Hotelling’s ellipses for each class. Green: non-toxic; blue: oxidative stress; red: phospholipidosis; purple; steatosis. Small spheres correspond to samples used to develop the model. Larger spheres correspond to the PLS-DA projection of the external validation samples, denoted as test samples and colored based on their predicted class. (**b)** Figures of merit of the PLS-DA model.

A PLS-DA model was built to evaluate whether the discovered metabolomic fingerprints were able not only to detect hepatotoxicity but also to classify the toxic effect according to its main mechanism of action (i.e. oxidative stress, phospholipidosis and steatosis). For model development and validation samples were split into two subsets, one for model development, using 80% of the samples and a different one for external model validation, which was set by a random selection of 20% of the samples equally distributed among the groups (Supplementary Table S4). To avoid redundant information and instrumental noise, MS data was submitted to a variable selection procedure^22^. Data reduction has been shown to be a straightforward strategy to achieve model simplification, and improve model handling and performance. Basically, the variable selection process consisted in ranking the variables (i.e., metabolites) attending to their VIP (variable importance in the projection) values obtained by the PLS-DA analysis. The final model was constructed using three latent variables and the top 26 ranked markers, which were selected according to the cross-validation results (Supplementary Figure S1, Supplementary Table S5). A good separation between the different groups, with almost no overlap between the 95% confidence Hotelling’s ellipses calculated for each class, was observed in the scores plot (Fig. 6a) and remarkable figures of merit were obtained by using cross validation (Fig. 6b). To further validate the PLS-DA model, two permutation tests were run, one using the misclassification error as the evaluating parameter, and the other using AUROC (area under the receiver operating characteristic curve) (Supplementary Figure S2). In both cases, the 95% confidence intervals of the values obtained with real assignments were beyond the values obtained using randomly permuted assignments. As the permutation tests were performed with 1000 permuted models, it was possible to assign an empirical *p* value of < 0.001^23^. Finally, a real model validation was performed by the assessment of an external set of samples, comprising samples that were not used for model building. The results reveal that all samples were correctly projected by the PLS-DA model with different degrees of confidence (Fig. 6a), thus strengthening its consistency.

## Discussion

The complexity of the mechanisms involved in hepatotoxicity and its unpredictable occurrence complicates the identification of drugs that have the potential to cause toxicity. Here, we intended to develop a cell-based metabolomic approach aimed not only to detect drug potential toxicity but also to classify its main mechanism(s) of action. HepG2 cells were chosen for the present study as they have been extensively used as cell model in hepatotoxicity testing^9^,^11^,^24^,^25^. Despite its functional and physiological differences with respect to hepatocytes, especially related to drug metabolism and transport, the HepG2 cell line meets several essential requirements that strengthens its utility for hepatotoxicity screening, including its human hepatic origin, widespread use, lifespan, easy handling and reproducibility^6^,^26^. One major drawback of these cells is their low metabolic capabilities. However, such limitation can be overcome by using different strategies such as transfection with adenovirus or addition of S9 fractions containing drug-metabolizing enzymes^27^. An important issue in the development of predictive models is the use of well-defined objects (model compounds) representative of each of the groups (toxicity mechanisms) that are to be studied; eventually, these compounds will be used to train the model and will define its consistency. Representative compounds of the three mechanisms of hepatotoxicity (i.e., steatosis, phospholipidosis and oxidative stress) were selected based on an exhaustive literature research and on our own data, all the model drugs can cause toxicity directly^11^,^28^. The simple experimental design consisted of 24 h of cells monolayers exposure to low drug concentrations (below or close to the IC10 value) at which toxic effects are observed in the absence of significant cell death. Such an approach allowed us to capture the initial specific effects of the drugs on cell metabolism/physiology as longer times or higher concentration exposures may lead to the appearance of non-specific damages or adaptation responses not directly related to the primary toxic effect. Nonetheless, drugs concentrations were below 100-fold their C_max_ (therapeutically active average plasma maximum concentration values upon single-dose administration at commonly recommended therapeutic doses), and thus the observed hepatotoxic effects are manifested at a concentration which is considered to be biologically relevant with respect to the one at which the liver is exposed upon administration of therapeutic doses^29^. To ensure a wide metabolomic coverage, cell extracts were analyzed by a metabolite profiling approach that provides the detection of both polar and lipid compounds^20^,^21^.

The rich holistic metabolomic MS data generated was subjected to both non-supervised (i.e. PCA) and supervised (i.e. PLS-DA) multivariate data analysis in a search of discriminant metabolomic patterns contributing to either generic or mechanism-specific hepatotoxic effects (Fig. 1). The results show that the analytical strategy succeeded in accomplishing both objectives, i.e. not only were gross/common metabolomic patterns causative of non-specific toxicity detected, but slight subtle mechanism-sensitive changes were appreciated (Fig. 1f,g). To deepen the knowledge on the alterations caused by each specific group of hepatotoxicants, pairwise comparisons (i.e. control vs. each hepatotoxicity mechanism) were performed (Fig. 2), which led to the identification of specific markers related to each toxicity mechanism (Supplementary Tables S2 and S3).

Redox homeostasis disruption is a common effect in many drug-induced adverse effects. The GSH/GSSG pool is the principal redox buffer within the cell^30^, and changes in this ratio are associated with early oxidative damage events^31^. A low GSH/GSSG ratio was observed in all the mechanisms (Fig. 3). Accordingly, high levels of CSSG, a marker of oxidative damage^32^, and other metabolites involved in GSH synthesis and recycling via the γ-glutamyl cycle (i.e. γ-glutamyl-glutamate, γ-glutamyl-glutamine, glutamate and glutamine), were also observed in the case of drugs exerting their toxicity via oxidative stress (Fig. 3). A targeted analysis revealed a dose-dependent decrease in the GSH/GSSG ratio for all hepatotoxic drugs that was accompanied by a dose-dependent increase in the levels of ophthalmic acid (Supplementary Figure S3), a non-sulfur-containing analog of GSH that has been proposed as a marker of oxidative stress and GSH depletion^33^. The abovementioned changes, in conjunction with the altered levels of acylcarnitines, FA and TG, point to mitochondria as the primary target of drug-induced damage. The oxidative processes that take place in mitochondria (along with the presence of unprotected mtDNA) make them sensitive targets of oxidative damage^34^. A direct consequence of mitochondrial damage is the impairment of those metabolic pathways that take place in the mitochondria, including FA β-oxidation. This situation results in the accumulation of FA and intermediates of FA oxidation such as acylcarnitines. FA can either be esterified into TG or remain in its free form; the latter contributes to mitochondrial dysfunction and increases oxidative stress^34^. Moreover, FA β-oxidation inhibition can lead to reduced ATP levels, thus impairing GSH biosynthesis, which is produced from glutamate, cysteine and glycine in two ATP-dependent steps (Fig. 3).

The liver plays a key role in fat metabolism, and excessive drug-induced lipid accumulation may provoke important lesions. Drug-induced steatosis is often reversible, but the presence of certain drugs may exacerbate or precipitate its progression to more severe conditions as steatohepatitis and cirrhosis. The accumulation of TG inside hepatic cells is the hallmark of hepatic steatosis. Different mechanisms leading to drug-induced hepatic steatosis have been identified; among them, the impairment of FA β-oxidation due to mitochondrial disturbances is of special relevance^28^,^35^. There is substantial evidence that FA can directly cause toxicity by increasing oxidative stress and through the activation of inflammatory pathways^36^, therefore, the accumulation of FA as TG is thought to be an adaptive and protective response of hepatocytes to excessive availability of free FA in the liver and its associated liver toxicity^37^. Accordingly, increased levels of DG, which suggest an increased flux of FA to TG synthesis, were also observed (Fig. 5). The increased levels of DG and phosphocholine could lead to higher rates of phosphatidylcholine biosynthesis via the CDP-choline pathway (i.e. Kennedy pathway)^38^,^39^, thus resulting in increased levels of PL, as observed (Fig. 4). Impairment of FA β-oxidation by steatogenic drugs can lead to an enhancement of extramitochondrial FA oxidation, thus promoting higher rates of ROS production and lipid peroxidation^40^. Oxidative stress has been shown to be an early event after lipid accumulation in the liver in patients with steatosis^41^. Accordingly, a decrease in GSH levels and the GSH/GSSG ratio as well as an increase in CSSG were observed in HepG2 cells exposed to steatosis-inducing drugs (Fig. 3). To further assess the biological relevance of these results, the changes induced in the hepatic metabolome of rats treated with a steatosis-inducing drug (i.e. tetracycline) were evaluated (Supplementary Figure S4). A total of 98 metabolites were found to be altered in the liver of rats as a result of the treatment, eight of which were common to those obtained in HepG2 cells (Supplementary Figure S5). Interestingly, despite the biological differences between the two experimental models, most of the metabolites that showed similar trends and significance were among the top-ranked discriminant variables (Supplementary Table S6) and similar trends were also observed with respect to the changes in the main classes of lipids (Supplementary Table S7). Overall, the results pointed to a similar mechanism of toxicity although the contributions of particular factors (i.e. oxidative damage) may differ between the biological models.

Over 50 marketed drugs have been reported to induce phospholipidosis in different tissues, including the liver^35^. Although drug-induced phospholipidosis is often reversible and there is no definitive evidence for its toxicological implications, it is considered an adverse side finding by regulatory agencies. The most characteristic alteration associated with phospholipidosis is the excessive accumulation of PL; however, this hallmark was not reproduced under our experimental conditions (24 h incubation with sub-lethal concentrations of the drugs) and only slight but non-significant increases in PL were observed in HepG2 cells. Phospholipidosis is a chronic process and PL accumulation in the liver is only observed after long-term/repeated treatment with the drug^35^. The observed decrease in LysoPL and in the LysoPL/PL ratio can be interpreted as an inhibition of the degradation of PL that would ultimately lead to excessive accumulation (Fig. 4). Indeed, impaired PL degradation by lysosomal phospholipases seems to be the principal mechanism of this process^35^,^42^. Although the mechanism responsible for this inhibition is unknown, it has been suggested that the drug can bind to PL, thus resulting in the formation of complexes that are either resistant to breakdown or can act as enzyme (phospholipase) inhibitors^43^. As an alternative mechanism, the accumulation of phospholipidosis-inducing drugs (usually cations) can neutralize the anionic surface charge in lysosomal lipid bilayers required for phospholipase activity^44^. Other studies have suggested that not only impaired phospholipase activity, but also alterations in lysosomal enzyme transport and PL or cholesterol biosynthesis are the mechanisms likely involved in the development of drug-induced phospholipidosis^24^. Besides the alterations in PL and LysoPL levels, increases in TG and OS markers were also observed in HepG2 cells treated with phospholipidosis-inducing drugs (Figs 3 and 5). It is known that some drugs that cause phospholipidosis, especially cationic amphiphilic drugs (CADs), can also induce mitochondrial damage. The complex structure and physicochemical characteristics of mitochondria (e.g., double membrane, mitochondrial membrane potential) facilitate the progressive accumulation of CADs, thus impairing their function^35^,^45^, which could lead to the induction of oxidative stress and steatosis^46^.

Transcriptomic-based analyses or cell imaging technology (i.e. high-content screening (HCS) have been applied to study the hepatotoxicity induced by drugs, including some of the compounds tested in our study^9^,^46^,^47^,^48^. Reasonable results have been reported (both in terms of sensitivity and specificity) when the aim is to differentiate between non-toxic and hepatotoxic compounds^9^ or even among drugs acting through a specific mechanism of hepatotoxicity as may be the case of steatosis^48^ or phospholipidosis^24^. However, only few studies have attempted to develop *in vitro* assays to classify drugs according to their main mechanism of toxicity^10^,^11^,^25^. A recent study revealed that transcriptomic profiles of HepG2 cells accurately classified known cholestasis-inducing drugs and non-hepatotoxic compounds, but predictions of other mechanisms of toxicity were not performed^25^. Other studies employed HCS alone or in combination with other mono-parametric tests^10^,^11^. HCS achieved highly satisfactory results, but for some compounds a high concentration was required (higher than the ones used in the present study and above 100x Cmax) to obtain a positive result. Moreover, in comparison to metabolic profiling HCS can provide only information about a few cellular parameters that need to be previously set up and require more than a single assay (assayed in parallel cells) to explore different mechanisms of drug-induced hepatotoxicity^11^,^48^. One of the key advantages of using holistic approaches is that they report a more comprehensive molecular snapshot of the systems under study. Particularly, metabolomics offers the closest *“omics”* analysis of cell phenotype, which provides valuable information about early toxic events and how they trigger subsequent changes in cell metabolic pathways. Obtaining wide metabolome coverage is an issue of special relevance to obtain the broadest overview of the system/situation under study. One key issue in the development of predictive models is the selection of the minimum number of variables (metabolites) that accomplishes a desired level of performance in terms of prediction or explanation; that is, the most parsimonious model. Data reduction helps in model simplification, noise elimination and the maximization of inter-group differences. Furthermore, the use of a low number of variables facilitates the later development of target quantitative analysis of the identified discriminating variables. This strategy was followed to develop a PLS-DA model with 26 variables, which was able to classify toxic drugs according to their main mechanism of hepatotoxicity, showing outstanding figures of merit (Fig. 6). The model was first mathematically evaluated by cross validation and class permutation testing, and showed proper robustness and consistency. To circumvent overoptimistic results due to the use of the same samples for model building and validation, the model was further evaluated by the use of an external validation set of samples, showing excellent results as all the samples used were correctly assigned with different degrees of confidence.

In summary, our main goal was to show that MS-based metabolite profiling can become a valuable tool to classify and investigate mechanism-specific hepatotoxic responses induced by drugs in a liver-derived cell model. Unique metabolomic fingerprints associated with oxidative damage, steatosis, and phospholipidosis were deciphered and used to develop a model, which was able to screen and classify hepatotoxicity based on the toxicants mode of action, even in the absence of cell death. The development of fast, quantitative and targeted analysis of the markers should increase throughput and minimize sample requirements, thus consolidating the incorporation of targeted metabolomics into the pre-clinical testing framework. This approach also allowed us to gain new insights into the molecular events underlying hepatotoxicity and to suggest toxicity-related pathways. These results need to be further confirmed using a larger number of compounds, and other *in vitro*/*in vivo* models should be tested for extrapolation to humans. In the near future, the approach described here could become a routine tool in early drug development for hepatotoxicity screening, helping researchers to understand the mechanisms underlying drug-induced liver damage, which may eventually lead to the development of safer drugs.

## Materials and Methods

### Materials

All LC solvents (i.e. water, methanol, acetonitrile and isopropanol) were of LC-MS grade and were purchased from Fisher Scientific (Loughborough, U.K.). All LC-MS additives (i.e. formic acid and ammonium acetate) and the analytical standards (when available) were acquired from Sigma-Aldrich (Madrid, Spain). Lithocholic acid-D4 (LCA-D4) was obtained from Steraloids (Newport, USA). Phenylalanine-D5 (Phe-D5) was purchased from Cambridge Isotope Laboratories (Tewksbury, USA).

### Culture and treatment of HepG2 cells

HepG2 cells were routinely grown in culture grade flasks at 37 °C under a humidified atmosphere 5% CO_2_/95% air in Ham’s F-12/Leibovitz L-15 (1:1, v/v) supplemented with 7% fetal bovine serum, 50 U/mL penicillin and 50 μg/mL streptomycin. The medium was renewed every 2 days. Cells reaching 70–80% confluence were ready to be used or passaged. For subculturing purposes, cells were detached by treatment with 0.25% trypsin/0.02% EDTA at 37 °C^49^. For the metabolomic studies, cells were seeded at a density of 8 × 10^4^ cells/cm^2^ on 6-well culture dishes.

Ten compounds were selected as model hepatotoxicity inducers based on the data available in the literature^28^ (Table 1). Two compounds with no reports of hepatotoxicity were used as the negative controls. The stock solutions of the test compounds were prepared in DMSO and were freshly diluted in the culture medium to obtain the desired final concentration. The final DMSO concentration in the culture medium never exceeded 0.5% (v/v). Two additional controls were employed (i.e. culture medium and DMSO 0.5% (v/v) in culture medium). Only sublethal concentrations of the compounds were used^11^,^20^,^50^ (Table 1). HepG2 cells (70–80% confluence) were treated for 24 h with the compounds. Three biological replicates were employed for each condition.

### Cell processing and analysis using LC-MS-based untargeted metabolomics

Metabolomic analyses were performed in a Waters Acquity UPLC chromatograph hyphenated to a Waters Synapt HDMS Q-ToF mass spectrometer (Waters, UK). Cells were processed and analyzed by following a previously optimized analytical strategy^20^,^21^. In summary, different metabolome extractions were combined to obtain polar and nonpolar fractions, which were then analyzed separately by hydrophilic interaction liquid chromatography (HILIC) and reversed-phase (RP) liquid chromatographic techniques (see Supplementary Information).

### Quality assurance strategy

Blank samples and a pooled QC sample were employed to monitor LC-MS system performance. Blank samples were obtained by applying the extraction protocol over empty cultured plates and were employed to identify those background ions that were associated either with the extraction solvents or chromatographic separation (mobile phases plus column bleeding). The pooled QC sample was injected at the beginning of the analysis and intercalated every 10 study samples to assess instrument stability in terms of retention time, peak area and mass accuracy for each IS added to the QC samples. Study samples were analyzed in randomized order. The quality assurance strategy has been provided in detail elsewhere^41^.

### MS data preprocessing and metabolite identification

Data processing was performed using the MZMine v.2.9.1 free software^51^. Data were normalized according to both the response obtained by the IS added to each sample during the preparation process^22^,^41^ and the total amount of biological sample, assessed by the total amount of protein^16^,^20^,^21^. Metabolite identification was performed by the query of the exact mass of the detected features against online databases within a certain mass range (±10 ppm). The online databases used were: the Human Metabolome Database (HMDB)^52^, the LIPID MAPS-Nature Lipidomics Gateway^53^ and the Metlin database^54^. The identity of the metabolites of interest was confirmed by comparing the MS/MS spectra of the selected features with those of the proposed metabolites in online databases HMDB^52^, Metlin^54^ and MassBank^55^. The identities of the selected metabolites were further confirmed by using authentic standards whenever available. Only those features that matched a known metabolite identity were further submitted to the data analysis process. The degree of confidence in the identification was defined as specified by the Metabolomics Standards Initiative^56^. To perform the data analysis, all the information for a given sample (i.e. the information provided by the different analytical conditions) was joined to a single matrix, which comprised all the data available for each biological sample, and the mean value for the three biological replicates performed for each condition was calculated^22^. Each drug concentration was considered as an independent entity.

### Statistical analysis & data interpretation

All the statistical analyses and data plots were run with the free software R^57^. PCA was used to visualize the natural interrelationship among the samples, either all at once or by performing pairwise comparisons (i.e., control vs. each toxicity mechanism). Those features that met at least one of the following criteria were considered as discriminant: i) *q* value < 0.05, *p* value calculated using Mann Whitney test (for pairwise comparisons, i.e. control vs. each toxicity mechanism or control compounds vs. hepatotoxins) or ANOVA (analysis of variance, for multi-group comparisons, i.e. identification of mechanism-specific differences in which a 4-group comparison was performed) with the Benjamini-Hochberg (false discovery rate, FDR) correction for multiple testing; ii) VIP value >1.2, by PLS-DA modeling^58^. Pathway analysis tools were used for biological data interpretation and for toxicity-related pathways unraveling^59^,^60^. PLS-DA was employed to develop classificatory/predictive models based on the altered metabolomic patterns aimed to discriminate between non-hepatotoxic compounds and those acting through each of the abovementioned mechanisms of hepatotoxicity (Table 1). The quality of the PLS-DA models was verified by the typical cross-validation procedure by leaving one-fifth of samples out of each round. In each round, four-fifths of the data are used to train the PLS-DA model and the remaining one-fifth is used as test set, the procedure is repeated until all the samples have participated in the test set. Model parameters used to evaluate model performance were total Y explained variance (i.e., R^2^); predictable Y variation (i.e., Q^2^); prediction accuracy (evaluated as the misclassification error rate); and AUROC or multiclass AUROC^61^, based on the prediction, at each round, of the one-fifth of samples that are left out of model training. To further assess model consistency and performance, a response permutation test (n = 1000) was applied. In brief, permutation testing compares the original model’s goodness of fit with the values obtained after class randomization^23^. In the case of the development of the PLS-DA model aimed at the discrimination between the different mechanisms of hepatotoxicity the data was split into two different subsets, 80% of samples were utilized in model development, while 20% of the samples (equally distributed among classes) were reserved as external validation set (Supplementary Table S4). Thus, only the samples belonging to the model development set participated in model building and optimization. A variable selection procedure was implemented to find the most parsimonious model. To this end, variables were ranked according to their VIP value and PLS-DA models were built with increasing number of variables. The optimum number of variables was selected as the one providing the highest figures of merit. Model validation was performed both by the use of permutation testing and by the assessment of an external validation set of samples. The data analysis workflow is depicted in Supplementary Figure S6. In all the cases, data sets were log-transformed, mean-centered and unit-variance-scaled prior to multivariate data analysis.

## Additional Information

**How to cite this article**: García- Cañaveras, J. C. *et al*. A metabolomics cell-based approach for anticipating and investigating drug-induced liver injury. *Sci. Rep.* **6**, 27239; doi: 10.1038/srep27239 (2016).

## Supplementary Material

Supplementary Information

## Figures and Tables

**Table 1:** Mechanistic classification, cytotoxicity to HepG2 cells and selected concentrations of the compounds included in the metabolomic study.

Compound	Abbreviation	Therapeutic class	Mechanism^a^	IC_10_(μM)^b^	IC_50_ (μM)^b^	C_max_^c^ (μM)	Concentration^d^ (μM)
Citrate	Cit	Urinary alkalinizer	C	>1000		NA	500, 1000
Ketotifen	Ket	Antihistaminic	C	130 ± 50	400 ± 200	0.0014	50, 100
Cumene hydroperoxide	Cum		OS	480 ± 90	800 ± 200	NA	50, 100, 250
*tert*-Butyl hydroperoxide	Tert		OS	280 ± 40	590 ± 180	NA	50, 100, 250
Amiodarone	Am	Antiarrhythmic	P	26 ± 4	78 ± 13	2.2	5, 10, 20
Clozapine	Clo	Antipsychotic	P	41 ± 12	60 ± 10	1.09	10, 20
Fluoxetine	Fluo	Antidepressant	P	12 ± 2	43 ± 5	0.93	20
Tilorone	Til	Antiviral	P	14 ± 3	65 ± 7	-	5, 20
Tamoxifen	Tam	Antiestrogen	P	32 ± 2	57 ± 5	0.27	15
Doxycycline	Dox	Antibiotic	S	600 ± 200	2200 ± 500	8.77	250, 500
Tetracycline	Tet	Antibiotic	S	640 ± 180	1350 ± 150	14.2	50, 100, 200, 400
Valproate	Val	Anticonvulsant	S	8870 ± 1500	17800 ± 3000	481	2000, 4000, 8000

NA: not applicable.

^a^Major mechanism involved in hepatotoxicity induced by the compound: S, steatosis; P, phospholipidosis; OS, oxidative stress; C, non-hepatotoxic (control)^28^,^50^.

^b^IC10 and IC50: the compound concentration that leads to a reduction of 10% and 50%, respectively, in viability (MTT assay) of HepG2 cells after 24 h of treatment^14^,^20^,^50^.

^c^C_max_: Therapeutically active average plasma maximum concentration values upon single-dose administration at commonly recommended therapeutic doses^11^,^35^,^50^.

^d^Concentrations used in the metabolomic study.
