# Supplementary material for: A metabolomics cell-based approach for anticipating and investigating drug-induced liver injury
Source: Sci Rep. 2016 Jun 6;6:27239. doi: 10.1038/srep27239 (PMC4893700; doi:10.1038/srep27239)
Supplement: Supplementary Information [file srep27239-s1.pdf]

Supplementary Information

A metabolomics cell-based approach for anticipating and investigating drug-induced liver injury

Juan Carlos García- Cañaveras [1,2], José V. Castell [1,2], M. Teresa Donato[1,2] and Agustín Lahoz [1]

[1]Unidad de Hepatología Experimental, Instituto de Investigación Sanitaria - Fundación Hospital La Fe, Valencia, Spain. CIBERehd, Centro de Investigaciones Biomédicas en Red de Enfermedades Hepáticas y Digestivas, FIS, Spain.

[2]Departamento de Bioquímica y Biología Molecular, Facultad de Medicina, Universidad de Valencia, Spain

*To whom correspondence should be addressed. Agustín Lahoz. E-mail: agustin.lahoz@uv.es. Unidad de Hepatología Experimental, Instituto de Investigación Sanitaria-Fundación Hospital La Fe, Avda. Fernando Abril Martorell, 106, 46026, Valencia, Spain. Tel: 961246620 Fax: 961246620

Supplementary Materials and Methods

Cell processing for LC-MS untargeted metabolomic studies

Cells were processed according to a previously optimized protocol [1,2].

After treatments, the culture medium was removed by aspiration. The cell monolayer was washed once with 1 mL of cold PBS and immediately frozen by the addition of liquid N_2. At this point, plates were stored at -80ºC until further processing.

Metabolite extraction and cell detachment were simultaneously performed by scraping the cells with 800 µL of a water:methanol:chloroform (10:27:3) solution containing 0.375 µg/mL reserpine and 0.075 µg/mL sulfadimethoxine as internal standards (IS). The cell extract/suspension was transferred to a clean tube. The possible cellular rests present in the plate were recovered with 400 µL of the same extraction solution and pooled with the previous volume. The cellular extract/suspension was submitted to three freeze/thaw cycles (liquid N_2/room temperature) to facilitate cell disruption and metabolite extraction. At this point three different aliquots were taken and processed independently. A 50-µL aliquot was mixed with 100 µL of 0.66 N NaOH and was used for protein determination [3]. A 600-µL aliquot was submitted to liquid-liquid extraction with chloroform by the addition of 300 µL of water and 450 µL of chloroform containing 0.01 µg/mL terfenadine as an IS. After vortexing (3 x 10 s), samples were allowed to rest at -20ºC for 20 min and were centrifuged (10 min, 10000 g, 4ºC). The upper aqueous and lower organic phases were separately transferred to clean tubes and evaporated to dryness. The organic phase was resuspended in 75 µL of a methanol:chloroform (3:1) solution containing 0.5 µg/mL verapamil as IS and was analyzed by the lipidomic-RP ESI (+) approach. The aqueous phase was resuspended in 75 µL of acetonitrile:water (70:30) solution with the IS (40 µg/mL Phe-D5, 20µg/mL 8BrAMP and 10 µg/mL Val-Tyr-Val) and was analyzed by the HILIC approach in both the ESI (+) and ESI (-) modes. Finally, the rest of the cell extract volume was centrifuged (10 min, 10000 g, 4ºC), and the supernatant was transferred to a clean tube and evaporated to dryness. The residue was resuspended in 75 µL of methanol:water (1:1) solution containing 4 µg/mL LCA-D4 as the IS to be analyzed by the generic-RP ESI (-) approach. In all cases, the dry residue was stored at -80ºC until analyzed and, once resuspended, it was centrifuged (10000 g, 10 min, 4ºC) before transferring the clarified supernatant to a 96-well plate for its LC-MS untargeted analysis.

In vivo hepatotoxicity studies in rats

Animal handling

Six-week-old male OFA rats (200 – 240 g) were purchased from Charles River (Barcelona, Spain) and acclimatized to laboratory conditions for at least 7 days. Animals were housed in individual cages with woodchip bedding in a room maintained at 21 – 25 °C, 30 – 70% humidity and a 12 h light-dark cycle. Each animal was allowed free access to water and standard chow diet (Scientific Animal Food and Engineering, Augy, France).

Rats were divided into two different groups: i) Tetracycline (n=10); ii) Control (n=8). Tetracycline was administered orally at a dose of 2 g/Kg/day 0.5 % methylcellulose solution, control rats were

administered vehicle [4]. Treatment was repeated during 4 consecutive days and sample collection and euthanasia were carried out 24 h after the last administration.

Rats were anesthetized with sodium thiobarbital (0.1 g/kg). Livers were removed, rinsed in PBS, divided into small portions, flash-frozen in liquid N_2, and stored at -80 ºC until further processing. All the experimental protocols were approved by the Institutional Animal Ethics Committee.

Liver tissue processing for LC-MS untargeted metabolomic studies

Each frozen tissue sample (around 100 mg) was placed in a 2 mL tube containing CK14 ceramic beads and weighted. For each 100 mg of tissue, 650 µL of methanol:water (3:1) containing the IS reserpine (0.375 µg/mL) and sulfadimethoxine (0.075 µg/mL) were added. Then, tissue was homogenized twice for 25 s at 6,000 rpm at 4 ºC in a Precellys 24 Dual system. After centrifugation (3000 g, 5 min, 4°C), the supernatant was transferred to a clean tube. A second extraction was performed with 350 µL per 100 mg of tissue of the solvent. Finally, the two extraction supernatants were pooled and stored at -80 ºC until further processing.

A 100 µL aliquot was transferred to a clean tube and evaporated to dryness using a speedvac. The dry residue was stored at -80 ºC until analysis. The residue was resuspended in 100 µL of water:methanol (1:1) containing 4 µg/mL LCA-D4 as IS. After centrifugation (10 min, 10000g, 4 ºC), the clarified supernatant was transferred to a 96-well HPLC plate and analyzed using the generic-RP analysis conditions in ESI (-) mode.

A 200 µL aliquot was transferred to a clean tube and 100 µL of chloroform containing 0.01 µg/mL terfenadine as IS were added. After vortexing (3 x 10 s), samples were allowed to rest at -20 ºC for 20 min and centrifuged (10 min, 10000 g, 4 ºC). Each phase (the upper aqueous and the lower organic) was separately transferred to a clean tube and evaporated to dryness in a speedvac. The organic phase was resuspended in 100 µL of methanol:chloroform (3:1) containing 0.5 µg/mL verapamil as IS and analyzed using the lipidomic-RP approximation in ESI (+) mode. The aqueous phase was resuspended in 100 µL of acetonitrile:water (70:30) containing 40 µg/mL Phe-D5 and 10 µg/mL Val-Tyr-Val as IS and analyzed using the HILIC approximation in both ESI (+) and ESI (-) modes.

LC-MS untargeted metabolomic analysis

Metabolomic analyses were performed in a Waters Acquity UPLC chromatograph hyphenated to a Waters Synapt HDMS Q-ToF mass spectrometer (Waters, UK) by following a previously optimized analytical strategy [2]. The data station operating software used was Masslynx v4.1 (Waters, UK). A Lock Spray interface, which allowed the co-introduction of eluting analytes and a reference compound directly into the ion source, was used to maintain mass accuracy during sample acquisition. Leucine Enkephalin (*m/z* 556.2771 or 554.2615, in ESI (+) and ESI (-), respectively) prepared at 50 pg/mL in acetonitrile/water (1:1), plus 0.1% formic acid, was infused as the reference compound at a flow rate of 50 µL/min with an isocratic pump.

Three different LC conditions were used: i) generic-RP analysis; ii) lipidomic-RP analysis; and iii) HILIC analysis. In all cases, the temperatures of the column and the autosampler were set at 40°C and 4°C, respectively. The sample injection volume was 5 µL and the flow rate was set at 0.4 mL/min. The ESI conditions were as follows: capillary was set at 3.2 kV and 2.8 kV in the positive and negative modes,

respectively; cone voltage was set at 40 V; desolvation and source temperatures were set at 380ºC and 120ºC, respectively; the flow rates of the cone and nebulization gases were set at 50 L/h and 800 L/h, respectively. The same parameters were applied for the simultaneous MS and MS/MS analyses, with a collision energy ramp from 5 to 60 eV in the MS/MS channel.

Generic-RP untargeted analysis

LC separation was performed in an Acquity UPLC HSS T3 (1.7 µm, 2.1 × 100 mm; Waters) column. Eluent solutions were 0.1% formic acid in water (solvent A) and 0.1% formic acid in acetonitrile (solvent B). A 26-minute elution gradient was run as follows: for the first 2 min, eluent composition was set at 99.9% A and 0.1% B, which was linearly changed to 75% A and 25% B in 4 min; then the proportion of B was increased to 80% over the next 4 min, followed by a further increase to 90% B reached at min 12 and 100% B at min 17, and was maintained for 5.5 min. Finally, the initial conditions were recovered and maintained for 2 min for column conditioning. Mass detection was carried out in the MS scan mode from 50 to 1000 Da in ESI(-).

Lipidomic RP untargeted analysis

LC separation was conducted in an Acquity UPLC BEH C18 (1.7 µm, 2.1 × 100 mm; Waters) column. Eluent solutions were 0.1% formic acid ammonium acetate 10 mM in water (solvent A), and 0.1% formic acid and ammonium acetate 10 mM in acetonitrile:isopropanol (5:2) (solvent B). An 18-minute elution gradient was performed as follows: the initial eluent composition was set at 65% A and 35% B, which was linearly changed to 20% A and 80% B in 2 min; then the proportion of B was increased to reach 100% at min 9 and was maintained for 7 min. Finally, the initial conditions were recovered and maintained for 2 min for column conditioning. Mass detection was run in the MS scan mode from 200 to 1200 Da in ESI (+).

HILIC untargeted analysis

LC separation was performed in an Acquity UPLC BEH Amide (1.7 µm, 2.1 × 100 mm; Waters) column. Eluent solutions were acetonitrile (solvent A) and ammonium acetate pH 3 20 mM in water (solvent B). An 18-minute elution gradient was performed as follows: for the first 3 minutes, eluent composition was set at 95% A and 5% B, which was linearly changed to 75% A and 25% B in 6 min; then the proportion of B was increased to reach 65% at min 13 and was kept for 2 min. Finally, the initial conditions were recovered and maintained for 2.5 min for column conditioning. Mass detection was carried out in the MS scan mode from 50 to 1000 Da in both ESI (+) and ESI (-).

Targeted analysis of oxidative stress markers

Targeted analysis of oxidative stress markers was performed in a Waters Acquity UPLC chromatograph hyphenated to a Waters Xevo TQS mass spectrometer (Waters, UK) by following a previously described LC-MS/MS method [1]. HepG2 cells (70-80% confluence) were treated for 24 h with either control compounds (**Table 1**) or hepatotoxins (i.e. tert-butyl hydroperoxide, amiodarone and tetracycline) and processed following the protocol described by Carretero et al [1].

Supplementary tables

Supplementary Table S1. Coefficient of variation (expressed as percentage) for the retention time and the peak area of the IS compounds added to the QC samples in the different analytical conditions (n=10).

Analytical Condition	Internal Standard	m/z	RT (min)	RT CV(%)	Peak Area CV(%)
**Lipidomic-RP ESI(+)**	Terfenadine	472.3216	1.88	0.4	11.7
	Verapamil	455.2910	1.26	0.7	8.3
	Reserpine	609.2810	1.32	0.5	6.3
**Generic-RP ESI(-)**	Sulfadimethoxine	309.0658	4.53	0.14	12.3
	Lithocholic acid-2,2,4,4-D4	379.3150	8.10	0.18	12.3
	Reserpine	609.2810	5.90	0.10	7.8
**HILIC ESI(+)**	Sulfadimethoxine	311.0814	0.68	0.0	7.5
	Phenylalanine-D5	171.1182	7.10	0.6	6.9
	Val-TyrVal	380.2185	6.33	0.8	14.7
**HILIC ESI(-)**	Sulfadimethoxine	309.0658	0.68	0.6	9.4
	Phenylalanine-D5	169.1020	7.10	0.2	14.6
	Val-TyrVal	378.2029	6.33	0.5	12.4

Supplementary Table S2. Detailed information about all the altered metabolites based on the previously established criteria.

Class	Compound	KEGG ID	LoA	Detection mode	Adduct	RT	*m/z*	Error	OS	P	S
Aminoacid & related compounds	Alanine	C00041	1	Generic-RP ESI(-)	M-H	2.67	88.0399	5	0.81**	0.75**	0.66**
	Argininosuccinate	C03406	1	HILIC ESI(+)	M+H	11.19	291.1295	1	1.3	1.76*	4.68**
	Aspartate	C00049	1	HILIC ESI(-)	M-H	10.19	132.0299	2	0.64***	0.7**	1.41**
	Citrulline	C00327	1	HILIC ESI(-)	M-H	9.34	174.0875	5	1.07	1.12	2.25**
	Creatine	C00300	1	HILIC ESI(+)	M+H	7.99	132.0764	2	1.35	1.41*	1.29
	Cysteineglutathione disulfide	-	1	HILIC ESI(-)	M-H	11.49	425.0773	7	1.48	2.19***	2.46**
	Diacetylspermidine	-	1	HILIC ESI(+)	M+	5.58	230.1862	2	0.18**	0.48	1.49
	ɣ Glutamyl-Glutamate	C05282	1	HILIC ESI(-)	M-H	10.60	275.0873	4	1.21**	1	1
	ɣ-Glutamyl-Glutamine	C05283	1	HILIC ESI(-)	M-H	10.76	274.1034	3	2.48*	2.08***	1.27
	Glutamine	C00064	1	HILIC ESI(-)	M-H	8.86	145.0610	5	2.22	1.9**	1.18
	Glutathione	C00051	1	HILIC ESI(-)	M-H	10.18	306.0757	2	0.49	0.12**	0.92
	Methionine	C00073	1	HILIC ESI(-)	M-H	6.44	148.0434	2	0.99	0.96	1.62**
	N-Acetyl-L-tyrosine	C01657	3	HILIC ESI(-)	M-H	3.22	222.0766	2	1.39**	1.29*	1.11
	Ornithine	C00127	1	Generic-RP ESI(-)	M-H	0.58	131.0820	4	0.87	0.94	1.57**
	Phenylalanine	C00079	1	Generic-RP ESI(-)	M-H	2.61	164.0706	6	0.96	0.89*	1.53**
	Taurine	C00245	1	HILIC ESI(-)	M-H	6.72	124.0073	0	1.18**	1.03	1.14
	Threonine	C00188	1	HILIC ESI(-)	M-H	8.05	118.0506	3	0.98	0.97	1.48***
	Tryptophan	C00078	1	Generic-RP ESI(-)	M-H	2.89	203.0818	3	0.98	0.91	2.45***
	Valine	C00183	1	HILIC ESI(-)	M-H	6.65	116.0719	1	0.95	0.82	1.86**
Cofactor	FAD	C00016	3	HILIC ESI(+)	M+H	10.77	786.1668	3	1.3	1.55*	1.14
	Pantothenic acid	C00864	1	Generic-RP ESI(-)	M-H	2.69	218.1020	6	0.82**	0.82*	0.67**
Nucleobases & Nucleotides	Adenine	C00147	3	HILIC ESI(+)	M+H	7.92	136.0617	0	3.33***	3.5****	2.01***
	AMP	C00020	2	HILIC ESI(-)	M-H	9.77	346.0548	2	0.72**	0.61**	1.04
	CMP	C08429	2	HILIC ESI(-)	M-H	10.60	322.0434	3	0.71**	0.64**	0.9
	GDP	C00035	2	HILIC ESI(+)	M+H	11.40	444.0297	4	1.41	1.53**	1.15
	GMP	C00144	2	HILIC ESI(-)	M-H	10.68	362.0489	5	0.61***	0.61***	0.77
	UDP	C00015	2	HILIC ESI(-)	M-H	11.24	402.9935	3	0.66*	0.79	1.04
	UDP-glucuronic acid	C00167	2	HILIC ESI(-)	M-H	11.67	579.0249	3	0.81	0.75**	1.1
	UDP-N-acetylglucosamine	C00043	2	HILIC ESI(+)	M+H	11.10	608.0884	0	1.36	1.23	1.78**
	Xanthine	C00385	3	HILIC ESI(-)	M-H	3.72	151.0254	4	1.19	1.19	1.64**
Organic acid	Citric acid	C00158	3	Generic-RP ESI(-)	M-H	0.64	191.0187	5	0.77	0.73*	0.73
	Fumaric acid	C00122	3	Generic-RP ESI(-)	M-H	0.62	115.0033	3	0.84	0.79*	1.22

Class	Compound	KEGG ID	LoA	Detection mode	Adduct	RT	*m/z*	Error	OS	P	S
Phospholipid metabolism	Glycerol 3-phosphate	C00093	3	HILIC ESI(-)	M-H	9.50	171.0055	5	0.83	0.8	0.56**
	Glycerophosphocholine	C00670	1	HILIC ESI(+)	M+H	9.03	258.1099	0	1.17	1.43*	1.56
	Phosphocholine	C00588	1	HILIC ESI(+)	M+H	9.84	184.0733	0	5.36*	2.34***	8.25****
	Phosphodimethylethanolamine	-	3	HILIC ESI(-)	M-H	9.54	168.0424	4	1.32	0.76	7.16***
Sugar phosphate	6-Phosphogluconate	C00345	3	HILIC ESI(-)	M-H	11.26	275.0172	0	0.86	0.8	0.43****
	Hexose-bisphosphate	C01231	3	HILIC ESI(-)	M-H	11.81	338.9875	3	0.77**	0.75**	0.91
	Hexose-phosphate	C00092	3	HILIC ESI(-)	M-H	10.73	259.0214	4	0.93	0.83	0.63***
Acylcarnitine	Carnitine(2:0)	C02571	3	HILIC ESI(+)	M+H	6.43	204.1231	0	1.08	1.4*	0.76
	Carnitine(6:0)	-	3	HILIC ESI(+)	M+H	4.22	260.1859	1	1.46**	1.34**	4.66
	Carnitine(5:0)	-	3	HILIC ESI(+)	M+H	4.42	246.1709	3	1.7***	1.47**	0.67
	Carnitine(3:0)	C03017	3	HILIC ESI(+)	M+H	5.52	218.1390	1	2.88**	1.64	1.16
Ceramide	Cer(d34:0)	-	3	Lipidomic-RP ESI(+)	M+H	8.22	540.5344	1	0.9	1.27*	2.25
	Cer(d36:0)	-	3	Lipidomic-RP ESI(+)	M+H	8.88	568.5677	2	0.62***	1.09	2.27
	Cer(d40:0)	-	3	Lipidomic-RP ESI(+)	M+H	10.13	624.6305	2	0.63**	1.04	1.54
	Cer(d42:0)	-	3	Lipidomic-RP ESI(+)	M+H	10.77	652.6609	1	0.65***	0.97	1.62
Diacylglyceride	DG(34:1)	C00165	3	Lipidomic-RP ESI(+)	$M+NH_4$	9.00	612.5555	1	0.79**	0.91	0.8
	DG(38:4)	C00165	3	Lipidomic-RP ESI(+)	$M+NH_4$	8.82	662.5710	1	0.85	0.9	1.46**
	DG(38:5)	C00165	3	Lipidomic-RP ESI(+)	$M+NH_4$	8.19	660.5569	1	0.98	1.07	2.52**
	DG(40:9)	C00165	3	Lipidomic-RP ESI(+)	$M+NH_4$	6.26	680.5204	6	1.03	1.04	1.5***
	DG(42:11)	C00165	3	Lipidomic-RP ESI(+)	$M+NH_4$	7.76	704.5306	8	1.07	1.04	1.51***
	DG(42:9)	C00165	3	Lipidomic-RP ESI(+)	$M+NH_4$	7.12	708.5511	7	0.98	0.97	1.4**
Free fatty acid	FA(16:0)	C00249	3	Generic-RP ESI(-)	M-H	9.77	255.2328	0	0.93	0.99	1.31**
	FA(16:1)	C08362	3	Generic-RP ESI(-)	M-H	9.19	253.2167	2	0.76**	0.8*	0.77
	FA(18:1)	C00712	3	Generic-RP ESI(-)	M-H	9.85	281.2484	0	0.86	0.87*	0.97
	FA(20:3)	-	3	Generic-RP ESI(-)	M-H	9.68	305.2488	0	0.7***	0.72**	0.78
	FA(20:4)	C00219	3	Generic-RP ESI(-)	M-H	9.26	303.2324	1	0.66***	0.65***	0.81
	FA(22:6)	C06429	3	Generic-RP ESI(-)	M-H	9.13	327.2331	0	0.67***	0.69***	0.9
Lysophospholipid	LysoPC(16:0)	C04230	3	Lipidomic-RP ESI(+)	M+H	2.96	496.3409	2	0.81	0.86	1.33**
	LysoPC(16:1)	C04230	3	Lipidomic-RP ESI(+)	M+H	2.62	494.3267	5	0.69	0.65**	1.04
	LysoPC(18:0)	C04230	3	Lipidomic-RP ESI(+)	M+H	3.44	524.3724	2	0.75	0.94	1.49**
	LysoPC(18:1)	C04230	3	Lipidomic-RP ESI(+)	M+H	3.01	522.3539	2	0.6**	0.79	0.81
	LysoPC(18:2)	C04230	3	Lipidomic-RP ESI(+)	M+H	2.74	520.3385	2	0.64**	0.67**	0.92
	LysoPC(20:0)	C04230	3	Lipidomic-RP ESI(+)	M+H	4.14	552.4036	2	0.4*	0.76	0.73
	LysoPC(20:1)	C04230	3	Lipidomic-RP ESI(+)	M+H	3.49	550.3845	3	0.56**	0.85	0.73
	LysoPC(22:1)	C04230	3	Lipidomic-RP ESI(+)	M+H	4.15	578.4211	5	0.45*	0.96	0.59

Class	Compound	KEGG ID	LoA	Detection mode	Adduct	RT	*m/z*	Error	OS	P	S
Lysophospholipid	LysoPC(22:6)	C04230	3	Lipidomic-RP ESI(+)	M+H	2.63	568.3422	4	0.63**	0.66**	1.26
	LysoPC(24:0)	C04230	3	Lipidomic-RP ESI(+)	M+H	5.87	608.4617	5	0.55*	1.14	1.08
	LysoPE(16:1)	C04438	3	Generic-RP ESI(-)	M-H	7.34	450.2627	0	0.64	0.71*	0.76
	LysoPE(24:1)	C04438	3	Lipidomic-RP ESI(+)	M+H	5.05	564.4049	4	0.57**	0.59**	0.45**
Phospholipid	PC(28:0)	C00157	3	Lipidomic-RP ESI(+)	M+H	6.26	678.5033	5	1.06	0.89	1.48***
	PC(28:1)	C00157	3	Lipidomic-RP ESI(+)	M+H	5.45	676.4910	0	1.2	1.41*	1.68
	PC(30:0)	C00157	3	Lipidomic-RP ESI(+)	M+H	7.12	706.5335	6	1.02	0.99	1.5***
	PC(30:1)	C00157	3	Lipidomic-RP ESI(+)	M+H	6.32	704.5241	2	0.93	1.04	1.51***
	PC(32:1)	C00157	3	Lipidomic-RP ESI(+)	M+H	6.37	732.5524	1	1.06	0.98	1.58***
	PC(34:0)	C00157	3	Lipidomic-RP ESI(+)	M+H	7.94	762.6068	7	1.09	1.01	1.44**
	PC(34:1)	C00157	3	Lipidomic-RP ESI(+)	M+H	7.20	760.5764	10	1.15	1.12	1.67****
	PC(34:2)	C00157	3	Lipidomic-RP ESI(+)	M+H	7.20	758.5709	1	1.06	1.08	1.34***
	PC(36:0)	C00157	3	Lipidomic-RP ESI(+)	M+H	8.65	790.6238	10	1.02	1.07	1.57***
	PC(38:1)	C00157	3	Lipidomic-RP ESI(+)	M+H	8.57	816.6471	0	0.79**	0.9	1.04
	PC(38:2)	C00157	3	Lipidomic-RP ESI(+)	M+H	8.34	814.6273	5	0.89	1.02	1.23**
	PC(38:5)	C00157	3	Lipidomic-RP ESI(+)	M+H	7.04	808.5845	0	0.97	1.03	1.66****
	PC(38:6)	C00157	3	Lipidomic-RP ESI(+)	M+H	6.76	806.5725	3	0.94	0.91	2.11****
	PC(38:7)	C00157	3	Lipidomic-RP ESI(+)	M+H	5.99	804.5531	0	0.83	0.92	1.97***
	PC(40:7)	C00157	3	Lipidomic-RP ESI(+)	M+H	6.77	832.5858	0	0.85	0.91	1.94**
	PC(40:8)	C00157	3	Lipidomic-RP ESI(+)	M+H	6.15	830.5694	0	0.88	0.81**	1.77**
	PC(44:12)	C00157	3	Lipidomic-RP ESI(+)	M+H	5.63	878.5723	3	0.77**	0.9	1.94**
	PE(30:1)	C00350	3	Lipidomic-RP ESI(+)	M+H	6.50	662.4803	7	1.06	0.99	1.33**
	PE(32:0)	C00350	3	Lipidomic-RP ESI(+)	M+H	6.71	692.5271	6	1.07	1.02	1.63***
	PE(32:1)	C00350	3	Lipidomic-RP ESI(+)	M+H	5.88	690.5070	0	1.05	1.01	1.51***
	PE(34:2)	C00350	3	Lipidomic-RP ESI(+)	M+H	7.38	716.5258	4	1.08	1.13	1.43***
	PE(36:1)	C00350	3	Lipidomic-RP ESI(+)	M+H	7.57	746.5694	0	0.95	0.98	1.45****
	PE(36:2)	C00350	3	Lipidomic-RP ESI(+)	M+H	8.11	744.5597	7	1.11	1.09	1.35***
	PE(36:3)	C00350	3	Lipidomic-RP ESI(+)	M+H	7.47	742.5403	2	0.97	0.84**	1.21
	PE(36:4)	C00350	3	Lipidomic-RP ESI(+)	M+H	7.24	740.5204	2	1.07	1.06	1.57***
	PE(38:2)	C00350	3	Lipidomic-RP ESI(+)	M+H	7.55	772.5848	0	0.97	0.96	1.24**
	PE(38:3)	C00350	3	Lipidomic-RP ESI(+)	M+H	6.89	770.5729	4	0.95	0.99	1.32***
	PE(38:5)	C00350	3	Lipidomic-RP ESI(+)	M+H	7.25	766.5404	2	1.1	1.11	1.43***
	PE(40:5)	C00350	3	Lipidomic-RP ESI(+)	M+H	6.69	794.5726	3	0.95	0.95	1.75***
	PE(42:7)	C00350	3	Lipidomic-RP ESI(+)	M+H	7.65	818.5739	5	1.07	1.13**	1.41***
	PE(42:9)	C00350	3	Lipidomic-RP ESI(+)	M+H	7.74	814.5454	8	1	1.38*	1.98***

Class	Compound	KEGG ID	LoA	Detection mode	Adduct	RT	*m/z*	Error	OS	P	S
Sphingomyelin	SM(d30:1)	C00550	3	Lipidomic-RP ESI(+)	M+H	5.18	647.5128	0	0.81	0.82*	0.86
	SM(d32:1)	C00550	3	Lipidomic-RP ESI(+)	M+H	6.14	675.5438	0	0.85*	0.96	1.02
	SM(d32:2)	C00550	3	Lipidomic-RP ESI(+)	M+H	5.28	673.5298	2	0.86	0.75***	1.01
	SM(d36:2)	C00550	3	Lipidomic-RP ESI(+)	M+H	7.12	729.5922	2	0.93	0.94	1.52****
	SM(d38:1)	C00550	3	Lipidomic-RP ESI(+)	M+H	8.76	759.6346	3	0.76**	0.98	0.98
	SM(d44:0)	C00550	3	Lipidomic-RP ESI(+)	M+H	11.02	845.7453	2	1.43***	1.41***	2.04***
Triacylglyceride	TG(36:0)	C00422	3	Lipidomic-RP ESI(+)	$M+NH_4$	9.54	656.5826	0	1.24	0.9	5.04***
	TG(38:0)	C00422	3	Lipidomic-RP ESI(+)	$M+NH_4$	10.09	684.6152	2	1.32	0.98	6.08***
	TG(38:1)	C00422	3	Lipidomic-RP ESI(+)	$M+NH_4$	9.54	682.5953	3	1.84	1.29	11.5***
	TG(40:1)	C00422	3	Lipidomic-RP ESI(+)	$M+NH_4$	10.02	710.6264	4	1.88	1.3	10.37***
	TG(40:2)	C00422	3	Lipidomic-RP ESI(+)	$M+NH_4$	9.51	708.6082	7	2.17*	1.42*	15.47**
	TG(42:0)	C00422	3	Lipidomic-RP ESI(+)	$M+NH_4$	11.27	740.6839	10	1.67****	1.23	2.41****
	TG(42:2)	C00422	3	Lipidomic-RP ESI(+)	$M+NH_4$	9.98	736.6412	5	1.86*	1.44**	11.31***
	TG(44:0)	C00422	3	Lipidomic-RP ESI(+)	$M+NH_4$	12.13	768.7078	0	2.3****	1.86**	3.04****
	TG(44:1)	C00422	3	Lipidomic-RP ESI(+)	$M+NH_4$	11.17	766.6895	3	1.46***	1.18	2.39****
	TG(44:2)	C00422	3	Lipidomic-RP ESI(+)	$M+NH_4$	10.47	764.6791	3	1.76**	1.54***	4.28***
	TG(46:0)	C00422	3	Lipidomic-RP ESI(+)	$M+NH_4$	13.13	796.7451	7	3.98***	3.11*	5.42***
	TG(46:1)	C00422	3	Lipidomic-RP ESI(+)	$M+NH_4$	11.99	794.7212	2	1.55***	1.43**	2.15****
	TG(46:2)	C00422	3	Lipidomic-RP ESI(+)	$M+NH_4$	11.09	792.7074	0	1.34	1.39**	2.35****
	TG(46:4)	C00422	3	Lipidomic-RP ESI(+)	$M+NH_4$	9.51	788.6754	1	0.81**	1.06	0.93
	TG(48:0)	C00422	3	Lipidomic-RP ESI(+)	$M+NH_4$	14.28	824.7767	7	6.53***	4.12	7.87**
	TG(48:2)	C00422	3	Lipidomic-RP ESI(+)	$M+NH_4$	11.84	820.7446	6	1.71***	1.37	2.48****
	TG(48:3)	C00422	3	Lipidomic-RP ESI(+)	$M+NH_4$	11.00	818.7250	2	1.51**	1.49***	2.33***
	TG(50:1)	C00422	3	Lipidomic-RP ESI(+)	$M+NH_4$	14.10	850.7842	1	2.1****	1.12	2.75***
	TG(50:3)	C00422	3	Lipidomic-RP ESI(+)	$M+NH_4$	11.72	846.7561	1	1.62**	1.2	2.11**
	TG(50:4)	C00422	3	Lipidomic-RP ESI(+)	$M+NH_4$	11.03	844.7344	5	1.6***	1.43***	2.18****
	TG(50:8)	C00422	3	Lipidomic-RP ESI(+)	$M+NH_4$	9.39	836.6721	4	1.15	1.11	1.63***
	TG(52:4)	C00422	3	Lipidomic-RP ESI(+)	$M+NH_4$	11.76	872.7684	2	1.76***	1.44**	2.31***
	TG(52:5)	C00422	3	Lipidomic-RP ESI(+)	$M+NH_4$	11.37	870.7543	0	1.72***	1.57***	3.41****
	TG(52:6)	C00422	3	Lipidomic-RP ESI(+)	$M+NH_4$	11.02	868.7435	5	1.53***	1.42**	3.45****
	TG(52:7)	C00422	3	Lipidomic-RP ESI(+)	$M+NH_4$	10.40	866.7172	6	1.64***	1.42	3.79****
	TG(54:7)	C00422	3	Lipidomic-RP ESI(+)	$M+NH_4$	10.94	894.7488	6	1.61**	1.39	3.34****
	TG(54:8)	C00422	3	Lipidomic-RP ESI(+)	$M+NH_4$	10.35	892.7360	3	1.71***	1.5*	4.03****
	TG(56:7)	C00422	3	Lipidomic-RP ESI(+)	$M+NH_4$	11.66	922.7823	3	1.97***	1.45	3.69****
	TG(56:8)	C00422	3	Lipidomic-RP ESI(+)	$M+NH_4$	10.86	920.7704	0	1.68***	1.58**	3.3****

Class	Compound	KEGG ID	LoA	Detection mode	Adduct	RT	*m/z*	Error	OS	P	S
Triacylglyceride	TG(58:1)	C00422	3	Lipidomic-RP ESI(+)	$M+NH_4$	11.38	962.9126	1	1.61	0.8	2.08**
	TG(58:2)	C00422	3	Lipidomic-RP ESI(+)	$M+NH_4$	11.41	960.8920	3	1.6	0.85	1.99**
	TG(58:8)	C00422	3	Lipidomic-RP ESI(+)	$M+NH_4$	11.50	948.8034	2	1.82***	1.33	3.43***
	TG(58:9)	C00422	3	Lipidomic-RP ESI(+)	$M+NH_4$	10.88	946.7802	5	1.62***	1.4	3.36***
	TG(60:15)	C00422	3	Lipidomic-RP ESI(+)	$M+NH_4$	9.08	962.7174	6	0.71****	0.7****	1.44
	TG(60:8)	C00422	3	Lipidomic-RP ESI(+)	$M+NH_4$	11.99	976.8348	2	2.05****	1.94***	2.8***
	TG(62:15)	C00422	3	Lipidomic-RP ESI(+)	$M+NH_4$	9.68	990.7451	9	0.8***	0.74***	1.22
	TG(62:16)	C00422	3	Lipidomic-RP ESI(+)	$M+NH_4$	8.98	988.7282	10	0.73***	0.77**	1.14

LoA: Level of Assignment based on the criteria established by the Metabolomics Standard Initiative [5]; **Adduct**: corresponds to the adduct that provides the highest intensity; **RT**: Retention time in minutes; ***m/z***: corresponds to the value obtained for the adduct that provides the highest intensity; **Error**: absolute value of the difference, calculated in ppm, between the observed *m/z* and the theoretical one; **OS**: fold of change for the mean value obtained for the oxidative stress group with respect to the control group; **P**: fold of change for the mean value obtained for the phospholipidosis group with respect to the control group; **S**: fold of change for the mean value obtained for the steatosis group with respect to the control group. DG: diacylglyceride, FA: free fatty acid, LysoPC: lysophosphatidilcholine, LysoPE: lysophosphatidilethanolamine, PC: phosphatidylcholine, PE: phosphatidylethanolamine, SM: sphingomyelin, TG: triacylglyceride. The notation for lipids indicate the total number of carbons in the FA moieties and the total number of double bonds. *p* value calculated using the Mann Whitney test corrected for multiple testing by using FDR. *, q value > 0.05, VIP > 1.2; **, q value < 0.05; ***, q value < 0.01; ****, q value < 0.001.

Supplementary Table S3. Detailed information about the alterations induced in the lipidome.

	Oxidative Stress		Phospholipidosis		Steatosis	
	q value	*FOC*	*q value*	*FOC*	*q value*	*FOC*
**Acylcarnitines**	0.02	1.67	0.011	1.17	0.6	1.13
**FA**	0.018	0.75	0.011	0.73	0.011	0.77
**DG**	0.7	0.96	0.9	0.99	0.03	1.29
**TG**	0.008	1.46	0.05	1.25	0.0002	2.13
**LysoPL**	0.04	0.68	0.08	0.72	0.7	1.05
**PL**	1.0	0.97	0.2	1.09	0.004	1.40
**LysoPL/PL**	0.018	0.71	0.05	0.79	0.011	0.69

***q* value** *p* calculated using the Mann Whitney test and corrected for multiple testing by using FDR; **FOC**: fold of change calculated as the ratio between the mean value obtained for treated versus control samples. DG: diacylglyceride, FA: free fatty acid, LysoPL: lysophospholipid, PL: phospholipid, TG: triacylglyceride.

Supplementary Table S4. Information about the samples included in the model development data set and in the external validation data set.

Class	Model Development	External Validation
**Control**	C<br>DMSO<br>Cit500<br>Cit1000<br>Ket50	Ket100
**Oxidative Stress**	Cum50<br>Cum250<br>Tert50<br>Tert100<br>Tert250	Cum100
**Phospholipidosis**	Am10<br>Am20<br>Clo10<br>Fluo20<br>Tam15<br>Til5<br>Til20	Am5<br>Clo20
**Steatosis**	Dox250<br>Tet50<br>Tet100<br>Tet400<br>Val2000<br>Val4000<br>Val8000	Dox500<br>Tet200

See **Table 1** for detailed information regarding the characteristics and abbreviation correspondence of each condition.

Supplementary Table S5. Detailed information about the 26 variables selected to be included in the PLS-DA model aimed at the discrimination between HepG2 cells treated with either non-toxic (control) or toxic compounds belonging to either of the mechanisms of hepatotoxicity (i.e., oxidative stress, phospholipidosis, steatosis).

	**ANOVA**	**C-OS**	**C-P**	**C-S**	**OS-P**	**OS-S**	**P-S**
**Adenine**	$4x10^{-5}$	0.26***	0.25***	0.38***	0.97	1.50	1.54
**LysoPC(20:0)**	0.009	1.86*	0.90	1.09	0.48**	0.58*	1.21
**PC(44:1).**	0.005	1.46	0.61	0.65	0.42**	0.44**	1.06
**Glutathione**	0.007	3.76*	10.66**	1.06	2.84	0.28	0.10*
**LysoPC(22:1)**	0.012	1.57	0.64	1.16	0.41**	0.74	1.8
**GSH/GSSG**	$3x10^{-8}$	3.24***	4.14***	3.62***	1.28	1.12	0.88
**LysoPC(20:1)**	0.04	1.55	0.93	1.18	0.6*	0.76	1.28
**γ-Glutamyl-Glutamine**	0.015	0.39*	0.46*	0.66	1.2	1.71	1.43
**LysoPC(24:1)**	0.02	1.40	0.62	0.94	0.44**	0.67	1.51
**LysoPC(18:2)**	0.009	1.44*	1.36*	1.02	0.95	0.71*	0.75*
**LysoPC(24:0)**	0.017	1.47	0.69	0.78	0.47**	0.53*	1.14
**SM(d44:2)**	0.02	1.21	0.69	1.07	0.57*	0.88	1.55*
**FA(20:4)**	0.015	1.47	1.61**	1.36	1.1	0.93	0.84
**LysoPC(26:1)**	0.04	1.33	0.70	0.87	0.53*	0.65	1.23
**LysoPC(18:1)**	0.10	1.45	1.01	1.08	0.70	0.75	1.07
**FA(22:6)**	0.04	1.43	1.50*	1.19	1.05	0.83	0.79
**Phosphocholine**	$01.5x10^{-4}$	0.19**	0.38*	0.11***	2.00	0.60	0.30**
**Aspartate**	0.002	1.44	1.39	0.67	0.96	0.47**	0.49***
**LysoPC(22:6)**	0.003	1.45	1.31	0.75	0.9	0.51**	0.57**
**FAD**	0.05	0.68	0.54*	0.71	0.8	1.05	1.32
**SM(d32:2)**	0.03	1.14	1.35	0.97	1.19	0.86	0.72*
**SM(d34:0)**	0.03	1.08	0.76	0.79	0.71*	0.73	1.04
**Carnitine(5:0)**	0.005	0.57	0.65	1.34	1.14	2.35**	2.06**
**FA(20:3)**	0.06	1.41	1.49*	1.39	1.06	0.99	0.93
**AMP**	0.03	1.35	1.6	0.93	1.19	0.69	0.58*
**N-Acetyl-Tyrosine**	0.08	0.65	0.73	0.76	1.13	1.17	1.03

Each row corresponds to one of the variables included in the PLSDA model, variables are ordered according to their importance in the model. **ANOVA:** refers to the *q* value obtained for an analysis of variance test (*p* value corrected for multiple testing by using FDR). The rest of the column represent the fold of change and the p value, calculated using the Tukey HSD post-hoc test, for each pairwise comparison. **C**: control, **OS**: oxidative stress, **P**: phospholipidosis, **S**: steatosis. *, p value < 0.05; **, p value < 0.01; ***, p value < 0.001.

Supplementary Table S6. Common altered metabolites in HepG2 and Rat models as a result of drug-induced steatosis.

	HepG2			Rat		
**Metabolite**	*q value*	*FOC*	*VIP*	*q value*	*FOC*	*VIP*
**PE(34:2)**	0.02	1.43	1.31 (Q1)	0.007	1.50	1.64 (D1)
**PE(38:2)**	0.05	1.24	1.11 (Q2)	0.010	1.94	1.70 (D1)
**SM(d36:2)**	0.010	1.52	1.57 (Q1)	0.03	1.58	1.50 (Q1)
**TG(52:4)**	0.011	2.31	1.54 (Q1)	0.05	1.86	1.02 (Q2)
**TG(52:5)**	0.009	3.41	1.69 (D1)	0.05	2.06	0.76(Q2)
**TG(56:7)**	0.003	3.69	1.71 (D1)	0.011	3.35	1.89 (D1)
**TG(56:8)**	0.010	3.30	1.65 (D1)	0.007	3.74	1.72 (D1)
**TG(60:8)**	0.02	2.80	1.42 (Q1)	0.004	3.14	1.94 (D1)

***q* value**: *p* value calculated using the Mann Whitney test corrected for multiple testing by using FDR; **FOC**: fold of change calculated as the ratio between the mean value obtained for treated versus control samples; **VIP**: variable importance in the projection value and ranking, in parenthesis, calculated for pairwise PLSDA models between control and steatosis samples. D1, Q1, Q2, variables ranked in the first decile, first quartile or second quartile, respectively.

Supplementary Table S7. Comparison of the changes induces in different classes of lipids in HepG2 cells and the liver of rat as a result of drug-induced steatosis.

	**HepG2**		**Rat**	
	q value	*FOC*	*q value*	*FOC*
**Acylcarnitines**	0.6	1.13	0.8	1.23
**FA**	0.012	0.77	0.12	0.79
**DG**	0.03	1.29	0.9	0.99
**TG**	0.0002	2.13	0.12	1.55
**LysoPL**	0.7	1.05	0.7	0.91
**PL**	0.003	1.4	0.8	0.97

***q* value** *p* value calculated using the Mann Whitney test corrected for multiple testing by using FDR; **FOC**: fold of change calculated as the ratio between the mean value obtained for treated versus control samples. DG: diacylglyceride, FA: free fatty acid, LysoPL: lysophospholipid, PL: phospholipid, TG: triacylglyceride.

Supplementary Figures

a

LV	R^2	Q^2	Error	AUROC
**1**	0.263	0.218	0.49±0.11	0.81±0.07
**2**	0.540	0.379	0.27±0.13	0.90±0.04
**3**	0.740	0.507	0.18±0.15	0.92±0.06
**4**	0.820	0.511	0.19±0.14	0.92±0.07

b

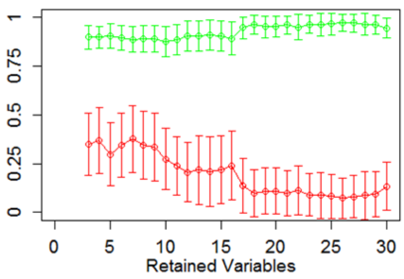

Supplementary Figure S1. Optimization of the number of latent variables (LV) and retained variables for the final PLS-DA model. a) Values of R^2 (blue), Q^2 (red) and misclassification error (green) as a function of the number of LV employed to build the PLS-DA model using all the variables. Based on the results, the optimum number of LV is set to 3. **b)** Values obtained for the AUROC (green) and misclassification error (red) vs the number of retained variables for the top-30 ranked variables using PLS-DA models with 3 LV. The data is expressed as mean ± standard deviation. Based on the results, the optimum number retained variables is set to 26. In both cases the PLS-DA models were aimed at the discrimination between HepG2 cells treated with either non-toxic (control) or toxic compounds belonging to either of the mechanisms of hepatotoxicity (i.e., oxidative stress, phospholipidosis, steatosis) using the model development data subset. The values were obtained based on cross-validation.

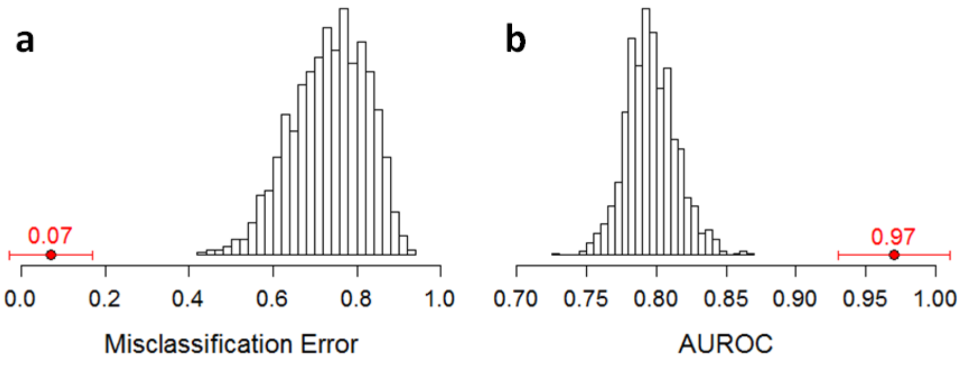

Supplementary Figure S2. Permutation tests validation for the final PLS-DA model. Permutation test for the misclassification error (**a**) and the multiclass AUROC (**b**) for the PLS-DA model built using 3 LVs and the top-26 ranked and aimed at the discrimination between HepG2 cells treated with either non-toxic (control) or toxic compounds belonging to either of the mechanisms of hepatotoxicity (i.e., oxidative stress, phospholipidosis, steatosis) using the model development data set (depicted in **Figure 46**). In both cases the histograms represent the values obtained using the permuted classes. The value obtained using the real classes is represented in red as mean ± standard deviation.

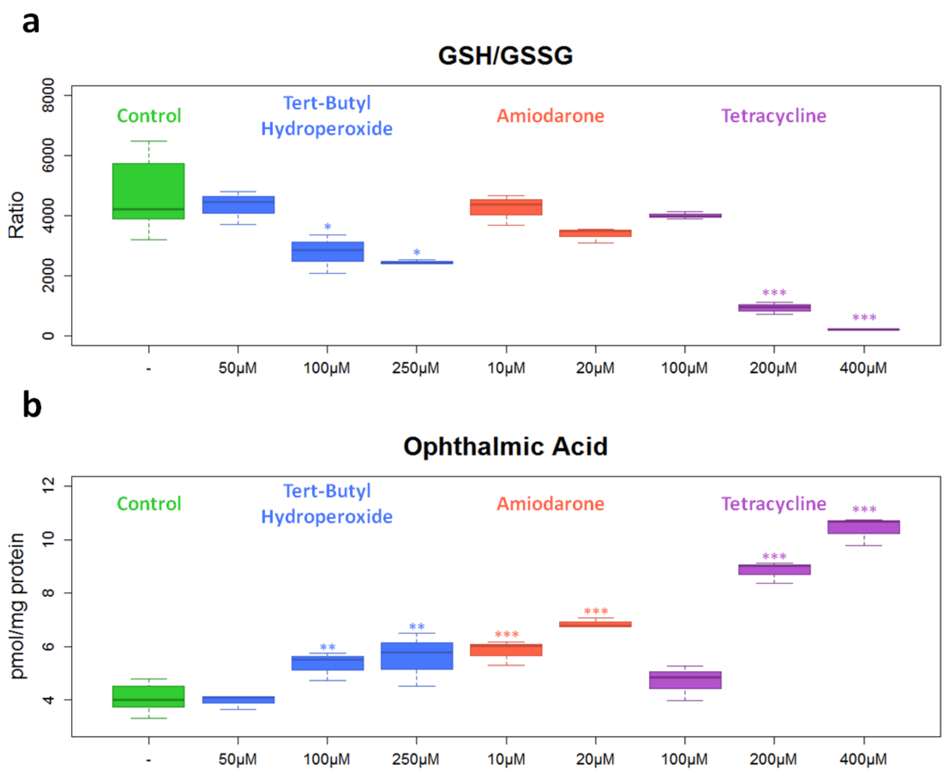

Supplementary Figure S3. Targeted analysis of oxidative stress markers. Boxplots showing the results obtained with the targeted analysis of OS markers (**a**, GSH/GSSG ratio; **b**, Ophthalmic acid) for HepG2 cells treated with control compounds (green), tert-butyl hydroperoxide (oxidative stress, blue), amiodarone (phospholipidosis, red) and tetracycline (steatosis, purple). Boxes denote interquantile ranges, lines denote medians, and whiskers denote the 10th and 90th percentiles. The value in the x-axis denotes the concentration at which the corresponding compounds has been tested. *, *p* value < 0.05; **, *p* value < 0.01; ***, *p* value < 0.001 calculated using the Mann Whitney test.

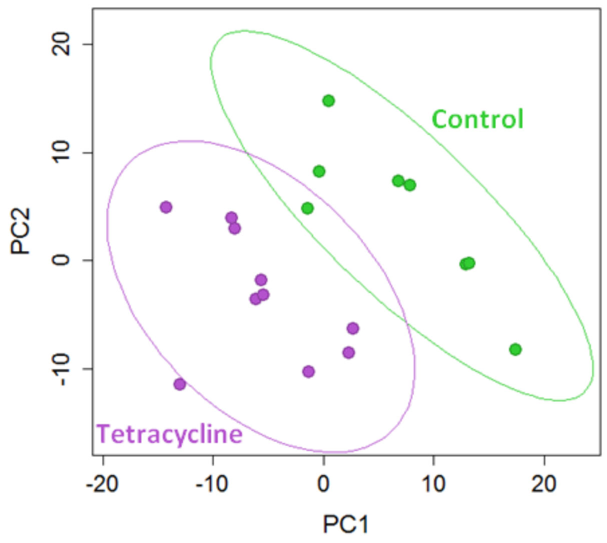

Supplementary Figure S4. PCA scores plots corresponding to data obtained from liver tissue of rats administered either vehicle (n=8) or tetracycline (n=10). Each point summarizes all the information provided by the four different analytical conditions. The lines denote 95% confidence interval Hotelling's ellipse. Green: control; purple: tetracycline

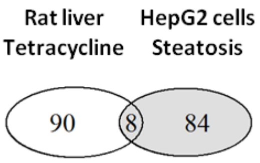

Supplementary Figure S5. Comparison between the steatosis markers obtained in rat liver and HepG2 cells. Venn diagram showing the overlap between the metabolites found to be altered as a result of tetracycline-induced toxicity in the liver of rats and of drug-induced steatosis in HepG2 cells.

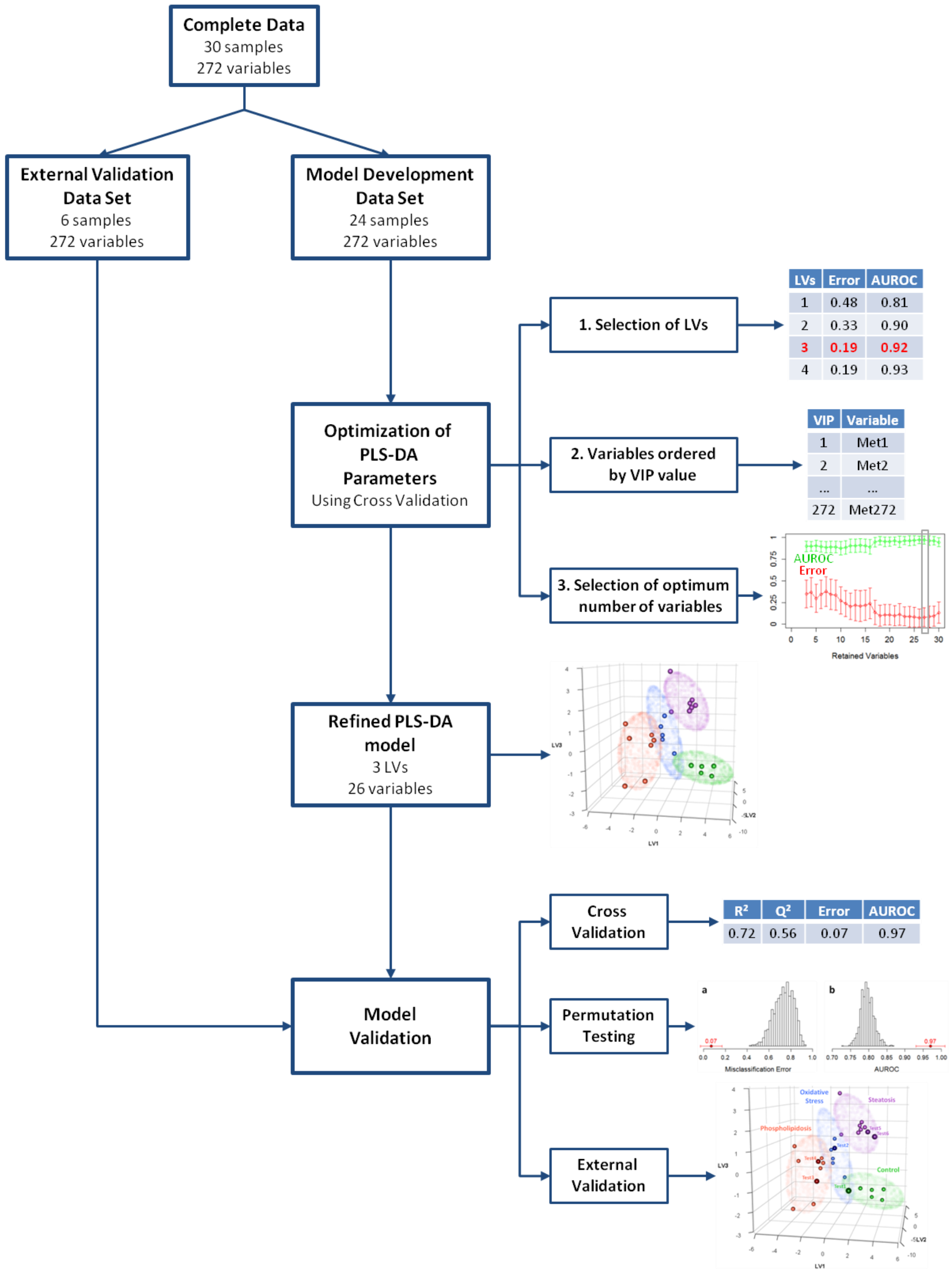

Supplementary Figure S6. PLS-DA modeling strategy. The complete data set is composed of 30 samples (belonging to 4 different classes) and 272 variables. The data is split into model development data set (24 samples) and external validation data set (6 samples equally distributed among classes) (**Supplementary Table S4**). The PLS-DA model is developed (and the parameters optimized) using only the model developent data set. Optimization of PLS-DA parameters is performed via cross validation (CV). First the number of latent variables (LVs) is set to that providing the best performance (3LVs). Then, the variables are ranked according to their VIP value and PLS-DA models with an increasing number of variables are built and their performance evaluated using cross validation. The optimum number of variables in the PLS-DA model is set to that providing the highest figures of merit (n=26). Thus based on the optimized paramenters a PLS-DA model using 3LVs and 26 retained variables is built. Model validation is performed using three different strategies: i) Cross validation, that allows to calculate

R^2, Q^2 and error and AUROC during the training procedure; ii) Permutation testing, which compares the goodness of fit of the actual PLS-DA model with respect to PLS-DA models built with permuted classes; and iii) External Validation, the samples belonging to the external validation data set are projected into the PLS-DA model and the accuracy of the prediction is calculated.

Supplementary References

1 Garcia-Canaveras, J. C., López, S., Castell, J. V., Donato, M. T. & Lahoz, A. Extending metabolome coverage for untargeted metabolite profiling of adherent cultured hepatic cells. *Anal Bioanal Chem*, doi:10.1007/s00216-015-9227-8 (2016).

2 García-Cañaveras, J. C. *et al.* LC-MS untargeted metabolomic analysis of drug-induced hepatotoxicity in HepG2 cells. *Electrophoresis* **36**, 2294-2302 (2015).

3 Gomez-Lechon, M. J. *et al.* Diclofenac induces apoptosis in hepatocytes. *Toxicol In Vitro* **17**, 675-680 (2003).

4 Kikkawa, R. *et al.* In vivo hepatotoxicity study of rats in comparison with in vitro hepatotoxicity screening system. *J Toxicol Sci* **31**, 23-34 (2006).

5 Sumner, L. W. *et al.* Proposed minimum reporting standards for chemical analysis Chemical Analysis Working Group (CAWG) Metabolomics Standards Initiative (MSI). *Metabolomics* **3**, 211-221 (2007).
